# Protein fractionation of *Hibiscus cannabinus* (kenaf) seeds, its characterization, and potential use for water treatment

**DOI:** 10.1002/wer.10805

**Published:** 2022-11-12

**Authors:** Benjamin U. Okoro, Soroosh Sharifi, Mike Jesson, John Bridgeman

**Affiliations:** ^1^ Department of Civil Engineering University of Birmingham Birmingham UK; ^2^ Department of Civil Engineering and Industrial Design University of Liverpool Liverpool UK

**Keywords:** coagulation, drinking water, nature‐based (plant‐based) coagulants, organic matter, water treatment

## Abstract

This study evaluates the coagulation performance of kenaf protein fractions (KPFs) comprising of albumin (AlbKP), globulin (GloKP), prolamin (ProKP), and glutenin (GluKP), in the treatment of high (500 NTU), medium (150 NTU), and low (30 NTU) turbidity water. Based on preliminary experimental results, the study focused on GloKP due to it outperforming the other kenaf coagulation products (KCPs) in all water types tested. The influence of GloKP, both as a primary coagulant and coagulant aid to aluminum sulfate (AS) on organic matter removal, was examined. Parametric analysis on turbidity, TSS, pH, dosages, retention time, and KPFs storage time was completed. Results indicated that GloKP could be used both as a primary coagulant and coagulant aid. GloKP had a higher turbidity and solids removal than the AlbKP and other KPFs (ProKP and GluKP). Solution pH greatly influenced the performance of the GloKP, and optimum dosage at pH 2 resulted in the highest organic matter removal. High dosages also resulted in negative mobility of particles and a more stable suspension. When used as a coagulant aid to AS, GloKP was more effective in removing dissolved organic carbon (DOC). Scanning electron microscopy elemental analysis (SEM‐EDAX) and Fourier transform infrared (FT‐IR) spectra showed the structure of the KPFs. SEM‐EDAX indicated the presence of metal cations capable of forming complexes essential for flocs formation. The enhanced floc formation, detailed in this paper, is ascribed to the collective effect of charge neutralization of the AS species and the adsorption and bridging effect of the GloKP, which improves the bonds formed between flocs. The coagulation–flocculation process can be significantly improved using dual coagulants. GloKP was also an excellent alternative to its crude (CrKP) and solvent extract (HxKP) form for removing suspended and dissolved particles from all water types.

**Practitioner Points:**

- Kenaf protein fractionates can destabilize stable particles.
- The globulin protein fractionate (GloKP) aggregated the most particles and contained least dissolved organic material.
- GloKP is pH sensitive with pH 2 reported as best working pH.
- Coagulant dosage and coagulation mechanism were assessed.

## Graphical abstract

Protein fractionated from kenaf seeds is a new promising anionic coagulant for water treatment. The most potent fraction, a globulin fraction—GloKP, effectively removed turbidity and dissolved organic carbon (DOC) when used as a coagulant aid. Monitoring of nutrient enrichment to treated water by the organic coagulants is recommended.

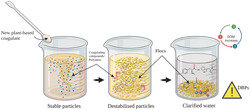

## INTRODUCTION

The use of chemical coagulants, including metallic coagulants, for example, aluminum and ferric salts, generally results in high particle removal in water treatment. However, their use results in the production of high volumes of sludge, for which advanced sludge disposal techniques are required; their production is energy intensive; and aluminum sulfate (AS) has been reportedly linked to Alzheimer's disease (Choudhary et al., 2019; McLachlan et al., 1996; World Health, 2003). These chemical coagulants are also prohibitively expensive for many people in developing countries, particularly in rural areas. Together, these limitations have resulted in increasing research interest in the use of natural coagulants, for example, plant‐based and animal‐based coagulants, and bio‐flocculants. A range of plant‐based coagulants and their coagulation products (PCPs) are used in water and wastewater treatment, especially in developing countries. This is partly due to their ease of cultivation and relatively low cost and also because they produce a low volume of sludge, are easy to use (with a broad range of operating pH and temperature), require only simple preparation and application during coagulation process, and are nontoxic (Choudhary et al., 2019). PCPs are readily available in most developing countries and often require very little preparation before use as a coagulant. One plant with good potential as a source for PCPs is kenaf (*Hibiscus cannabinus*), an annual crop found in Africa, China, India, and Thailand (Dempsey, 1975). Kenaf has a high protein content and contains compounds such as carbohydrate and phenols (Mariod et al., 2010; Mohamed et al., 1995), which give its particle destabilization ability.

A primary limitation reported during the use of these kenaf coagulation products (KCPs) is their contribution to the organic matter content of treated water (Okoro, Sharifi, Jesson, Bridgeman, & Moruzzi, 2021), which has cast doubts on their safe use for drinking water purification. Different treatment techniques have been researched to explore new ways to reduce the organic carbon content of these KCPs. These treatment methods include ion chromatography (Jones & Bridgeman, 2016) and ammonium sulfate precipitation (Arunkumar et al., 2019) and have been used to isolate different coagulating compounds present in these PCPs. These isolated compounds have excellent turbidity and organic matter removal rates (Gassenschmidt et al., 1995). Obtaining these isolated compounds is, however, expensive and requires specialized training and equipment, which is beyond the reach of those in the developing nations. A less expensive option of improving the quality of the KCPs could be the protein fractionation process described by Osborne and Voogt (1978), which involves the separation of their protein based on their solubility level. This protein separation process has been used to obtain isolates of some PCPs such as *Moringa oleifera* (Baptista et al., 2017) and *Hibiscus cannabinus* (kenaf) (Mariod et al., 2010). Based on the advantages of KCPs, combining metallic salt such as AS with KCPs has the potential of eradicating problems such as high sludge production and Alzheimer's disease, reportedly caused by using high dosages of AS (Choudhary et al., 2019).

The performance of these KCPs when used as a coagulant or coagulant aid for water treatment has been the subject of a small number of studies, and to the authors' knowledge, no studies to date have examined the performance of KPFs. Among the benefits recorded from the use of KCPs are improvement in their turbidity removal rate and floc properties (Jones & Bridgeman, 2016). However, very little information on their coagulation–flocculation mechanism is currently available. Recently, the protein from three Hibiscus plants, that is, okra, roselle, and kenaf, were isolated using ion chromatographic separation (ChrKP) and used both as a primary coagulant and coagulant aid (Jones & Bridgeman, 2016). Their use as an aid to AS (Al_2_(SO_4_)_3_) was shown to give rise to an improved floc strength and 64.3% higher regrowth rate. There has been no previous report on the coagulation–flocculation ability and mechanism of KPFs, so a detailed analysis, as presented in this paper, provides important new information and adds to the existing wealth of knowledge in turbidity and NOM removal from water systems.

This paper presents results that evidence the potential for KPFs use in point‐of‐use water treatment, especially in developing countries and small households. Following a description of the research methodology (Section 2), the first quantification of the performance of KPFs (albumin [AlbKP], globulin [GloKP], prolamin [ProKP], and glutenin [GluKP] fractions) are presented in Section 3. Section 4 then focuses on the best performing of the three KPFs and GloKP to provide information on its coagulation mechanism under a range of pH, dosage, and contact time. The paper closes with a summary of the key conclusions drawn from this research (Section 5).

## MATERIALS AND METHODS USED

The organic matter removal rate of the KPFs was measured using standard quality indicators: turbidity removal, light‐absorbing compounds at 254 nm (UV_254_), specific ultraviolet absorbance (SUVA_254_), and dissolved organic carbon (DOC). Experiments were performed for a range of pH values (pH 2 to 10) and dosages. UV_254_ and DOC are surrogate parameters of total organic carbon (TOC) concentration in the water while SUVA_254_ is a surrogate for NOM composition. The surface structure and morphology were determined using Fourier transform infrared (FT‐IR) technique and scanning electron microscope equipped with an energy dispersive X‐ray analyzer (SEM‐EDAX).

### Water samples

The source of water samples was the Bourn brook river, Birmingham, and samples were collected between March 2019 and October 2019. The turbidity value range is consistent with previous research (Okoro, Sharifi, Jesson, Bridgeman, & Moruzzi, 2021) and represents various turbidity types seen in surface water used in low‐income countries (Lantagne et al., 2007; Petersen et al., 2016; Wilhelm et al., 2018). Similar to the collection method used in Okoro, Sharifi, Jesson, Bridgeman, and Moruzzi (2021), low turbidity water (LTW) and medium turbidity water (MTW) were collected from two points along the river channel, while the high turbidity water (HTW) was derived by adding scooped river‐bottom sediment to the MTW. Only samples with turbidity within 10% of the target values of 30, 150, and 500 NTU were used. Water was collected in 10‐L containers and stored at 4°C to minimize changes to the physicochemical properties of water. While acknowledging that 5% is commonly used by researchers as a cutoff error, the sample size (80 samples) in the current study is small (although considered sufficient). So, it appeared reasonable to use 10% of target turbidity values (Stevens, 2012). Besides, the 10% is a widely adopted criterion in similar studies (Cao, 2015; Ma & Cao, 2019; Stevens, 2012).

### KPFs and water characterization

DOC measurements were performed using the TOC analyzer (Shimadzu TOC‐V‐CSH) with experiments performed at room temperature (19 ± 2°C). UV_254_ measurements were carried out in a 10‐cm‐high precision quartz cuvettes with a Varian Cary 50 Probe. A background spectrum using ultrapure water was obtained before analysis. The results obtained provided information on the concentration of organics and also helped in predicting the SUVA_254_ value, which defines the type of NOM present, as shown in Equation 1. SUVA_254_ values above four (4) indicate that organic compounds are predominantly hydrophobic and aromatic, while SUVA_254_ values less than three (3) describes that the organic materials are hydrophilic (Matilainen et al., 2011).

(1) $${\text{SUVA}}_{254}\;L/\mathrm{mg}-m=\frac{{\mathrm{UV}}_{254}\text{in}\;{\mathrm{cm}}^{-1}}{\mathrm{DOC}\text{in}\;\mathrm{mg}/L}\times 100$$

The Lowry method of protein determination (Lowry et al., 1951) was used to estimate protein concentration in the KPFs by taking readings of absorbance values at 660 nm (Table 1). Protein concentrations in the KPFs were used for turbidity experiment using a Turbidity meter (Hach 2100N), which followed the procedure of the Standard Method for Examination of Water and Wastewater (Rice et al., 2012). To improve understanding of the influence of pH on the system, NaOH and HCl were used in adjusting pH to 2.0–10.0. pH was determined using a pH meter (Thermo‐Scientific Orion 3 Star) while electrophoretic mobility (EM) was converted to zeta potentials by using the Smoluchowski equation. The Zeta‐Meter System was also used to measure the EM of the particles with a disposable capillary cell with gold‐plated electrodes (DTS 1070, Malvern Instrument, UK) and was expressed as microns/second per volt/centimeter (μcm s^−1^ V^−1^). EM measurements were conducted within 2 h of sample collection.

**TABLE 1:** Protein concentration of KPFs used during coagulation–flocculation experiments

Water type	KPFs	μg polymer/mg solid					
LTW	AlbKP	1.74 (0.13)	8.72 (0.64)	17.45 (1.28)	34.90 (2.57)	87.25 (6.42)	174.50 (13)
	GloKP	3.89 (0.29)	19.43 (1.43)	38.85 (2.86)	77.70 (5.72)	194.25 (14)	388.50 (29)
	ProKP	0.57 (0.04)	2.83 (0.21)	5.65 (0.42)	11.31 (0.83)	28.27 (2.08)	56.53 (4.16)
	GluKP	0.05 (0.003)	0.23 (0.02)	0.46 (0.03)	0.92 (0.07)	2.30 (0.17)	4.61 (0.34)
	AlbKP	0.37 (0.13)	1.86 (0.64)	3.72 (1.28)	7.45 (2.57)	18.61 (6.42)	37.23 (13)
MTW	GloKP	0.83 (0.29)	4.14 (1.43)	8.29 (2.86)	16.58 (5.72)	41.44 (14)	82.88 (29)
	ProKP	0.12 (0.04)	0.60 (0.21)	1.21 (0.42)	2.41 (0.83)	6.03 (2.08)	12.06 (4.16)
	GluKP	0.01 (0.003)	0.05 (0.02)	0.10 (0.03)	0.20 (0.07)	0.49 (0.17)	0.98 (0.34)
	dV (ml/L)	0.1	0.5	1	2	5	10
HTW	AlbKP	1.09 (1.28)	2.19 (2.57)	5.47 (6.42)	10.95 (13)	16.42 (19)	21.89 (26)
	GloKP	2.44 (2.86)	4.88 (5.72)	12.19 (14)	24.38 (29)	36.57 (43)	48.76 (57)
	ProKP	0.36 (0.42)	0.72 (0.84)	1.79 (2.10)	3.58 (4.2)	5.37 (6)	7.16 (8)
	GluKP	0.03 (0.03)	0.06 (0.07)	0.14 (0.17)	0.29 (0.34)	0.43 (0.51)	0.58 (0.68)
	dV (ml/L)	1	2	5	10	15	20

*Note*: dV, equivalent dosing volume measured into the beakers (ml/L); (*) values enclosed in bracket are protein concentration (mg/L) estimated using the Lowry method.

The Fourier transform infrared (FT‐IR) technique and the SEM‐EDAX were used to provide evidence of KPFs coagulation–flocculation ability. FT‐IR provided the chemical fingerprint and functional groups of KPFs, and the sludge generated from the treatment process. FT‐IR analysis was done using a PerkinElmer Fourier Transform Infrared spectrometer equipped with a deuterated tryglycine sulfate (DTGS) detector. Four scans and 8 cm^−1^ instrumental resolution were used to collect the spectrum from 4000 to 500 cm^−1^. A sample of the dried KPF was tested using a universal attenuated total reflectance accessory, following the manufacturer's instructions. The surface morphologies of dry KPFs and treated sludge samples were analyzed using scanning electron microscope (Hitachi TM3030 Tabletop SEM, Japan). The analysis was undertaken to visualize the surface structure of the samples. SEM samples were mounted on aluminum stubs by using double‐sided adhesive tape. The analysis involved using different resolutions and magnifications in examining the samples. The samples elemental analysis was qualitatively analyzed by EDX microanalysis.

### Collection and preparation of kenaf protein fractions

Air‐dried kenaf (*Hibiscus cannabinus*) seeds were obtained from a local market in Yola, Adamawa State, Nigeria. After collection, the dried kenaf seeds were pulverized using an electric grinder and then sieved through a 300‐μm steel sieve. Following Mariod et al. (2010) and Baptista et al. (2017), 20 g of the sieved seed (known as the crude extract, CrKP) was transferred to a cellulose thimble and defatted using analytical‐grade n‐hexane in a Soxhlet apparatus for 6 h (AOCS, 1998) at a solid/solvent ratio of 1:20. Then process lasted for 8 h with each complete cycle taking 2–3 min before the residue was collected and dried overnight at room temperature (19 ± 2°C). The KPFs were derived by using a modified Osborne fractionation process (Osborne & Voogt, 1978) to separate the water‐soluble (albumin), salt‐soluble (globulin), ethanol‐soluble (prolamin), and alkali‐soluble (glutenin) fractions of the defatted seed; 1% w/v of defatted flour was successively extracted with milli‐Q (ultrapure) water, 0.5 M NaCl solution, 1% w/v aqueous ethanol, and 0.1 M NaOH. After the extraction process, the suspension was centrifuged at 10,000 × *g* for 40 min, and then the supernatant and residual were carefully separated by decanting and vacuum filtering. The filtrate and decanted liquid were both dialyzed against ultrapure water in a dialysis tube (molecular weight cut‐off [mWCO] of 14 kDa; Spectra/Por Dialysis Membrane) at 4°C and for 24–36 h, and then the residue was used for the next extraction phase.

### Coagulation–flocculation experiments

The coagulation–flocculation experiments were conducted using a standard jar test apparatus (Phipps and Bird, UK), comprising of six 1‐L cylindrical beakers mixed using centrally coordinated, two‐blade flat paddle stirrers. For each of the coagulants and water types (HTW, MTW, and LTW), six different dosages (Table 1) selected from preliminary dosages trials were used to determine the optimum dosage required for the experiment. These dosages were selected during preliminary studies and represented dosages that are typically used in household water treatment and storage systems (HWTS). The best of these dosages corresponded to optimum turbidity at 70 min settling period. All coagulation–flocculation experiments started with a rapid mix of 200 rpm for 1 min and then a slow mix of 60 rpm for 15 min to allow for growth of flocs. Flocs formed above the 60‐rpm slow mix rate are usually disturbed and redispersed (Bulusu et al., 1968). So, the slow mix value used in this study suggests the extreme (worst) mix‐rate favorable for flocs formation in KCPs coagulated water. Results presented outlines the potentials of KCPs comparable to polyelectrolytes used in previous studies with slow mix ranging from 20 to 60 rpm (Bratby, 2016; Jones & Bridgeman, 2016; Muyibi & Evison, 1995; Okoro, Sharifi, Jesson, Bridgeman, & Moruzzi, 2021). After mixing was completed, samples were allowed to stand for 70 min before withdrawing unfiltered supernatant from 30 mm below the liquid surface with a syringe into the turbidimeter sample cell for measurements. The optimum dosage selected was then used for conducting turbidity experiment across a range of settling time (10, 20, 50, 70, 120, and 1440 min [24 h]).

The turbidity removal rate was calculated using the formula shown in Equation 2:

(2) $$\%\;\text{turbidity removal}=\frac{T_{\text{initial}}-T_{\text{residual}}}{T_{\text{initial}}}\times 100$$

where $T_{\text{initial}}$ is the turbidity value of the unfiltered untreated water and $T_{\text{residual}}$ represents the turbidity value of the treated water.

## RESULTS AND DISCUSSIONS

The percentage of proteins extracted from kenaf seed was verified to be 96.38% out of the total present (Table 2), leaving only a these extracted KPFs 3.21%. From Table 2, these extracted KPFs consisted of 36.85% AlbKP (water‐soluble fraction), 52.08% GloKP (salt soluble fraction), 3.21% ProKP (ethanol‐soluble fraction), and 0.93% GluKP (NaOH soluble fraction). The AlbKP and the GloKP were the largest fractions present in the kenaf seeds, consistent with previous findings of globulin being the predominant protein component in vegetable cotyledons (Baptista et al., 2015; Freitas et al., 2015).

**TABLE 2:** KPFs fraction percentages, zeta potential, and pH

KPFs	Fraction (%)	pH	Zeta potential (mV)
Kenaf	100	6.41	−15.21 ± 0.40
AlbKP	36.85	5.02	−10.11 ± 0.21
GloKP	52.08	6.07	−12.70 ± 1.50
ProKP	3.21	7.22	−22.31 ± 2.60
GluKP	0.93	12.80	−30.12 ± 0.17
Insoluble	3.31	11.03	−26.10 ± 2.80

*Note*: Results are obtained from means of three determinations ± standard deviation (for zeta potential).

pH and the zeta potential of the KPFs were obtained by making a solution of 1% w/v. As illustrated in Table 2, the pH values obtained are related to the type of solvent used during the extraction process. The values recorded for the zeta potential were all negative and is consistent with previous studies, which reported Hibiscus seeds as predominantly having negatively charged surfaces (Jones & Bridgeman, 2016; Okoro, Sharifi, Jesson, Bridgeman, & Moruzzi, 2021).

Untreated water used for this study had varying concentrations of quality indicators, as are shown in Table 3. MTW and HTW were moderately and highly enriched with dissolved and suspended particles. The test water also had high values of UV_254_ and SUVA_254_, which reflect organic material compounds present in water. The SUVA values indicate the specific absorbance at a particular wavelength and can be calculated using Equation 1. LTW gave the lowest SUVA_254_, DOC, and turbidity values signifying a reduced concentration of hydrophobic NOM. The zeta potential values showed that all tested water were negatively charged indicating that organic particles were mostly anionic, which agrees with previous report (Gassenschmidt et al., 1995). Similar charges of the suspended and dissolved organic matters results in the particles repelling each other, which gives rise to the stable and cloudy suspension mostly seen in untreated water.

**TABLE 3:** Turbidity, UV_254_, SUVA_254_, pH, zeta, and DOC values of the high, medium, and low turbidity water

Quality variable	HTW	MTW	LTW
Turbidity (NTU)	510 ± 02	150 ± 3.5	32 ± 3.4
UV_254nm_ (cm^−1^)	1.8 ± 0.2	0.7 ± 0.1	0.1 ± 0.02
SUVA_254_ (L/mg m)	14.8 ± 0.2	7.0 ± 0.9	1.7 ± 0.1
pH	7.2 ± 0.3	7.1 ± 0.1	7.1 ± 0.1
Zeta potential (mV)	−16.1 ± 0.6	−13.1 ± 1.07	−13.0 ± 0.1
DOC (mg/L)	12.1 ± 0.2	10.2 ± 0.2	8.0 ± 0.3

*Note*: Results are obtained from means of three determinations ± standard deviation; sample size = 80. Experimental condition: untreated water, T = 20°C.

### FT‐IR and SEM characterization of KPFs

**FIGURE 1:**
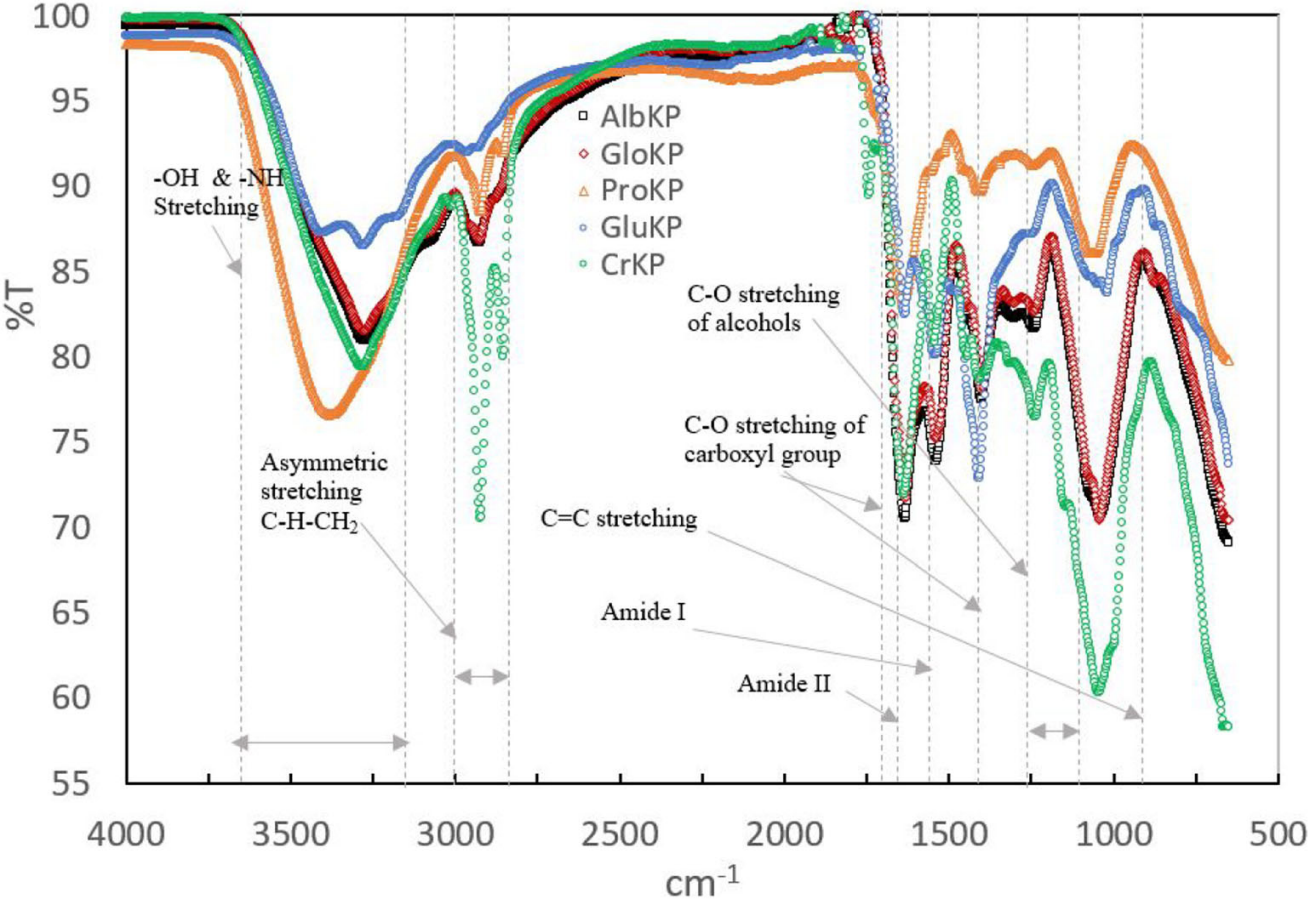
FT‐IR spectra showing AlbKP, GloKP, ProKP, and GluKP

Results from FT‐IR provided insight into the chemical composition and main functional groups present in the KPFs. From the results shown in Figure 1, the broad peak between 3300 and 3580 cm^−1^ indicates the presence of hydroxyl and amine groups, which form part of the compounds present in protein, carbohydrates, and fatty substances (Okoro, Sharifi, Jesson, Bridgeman, & Moruzzi, 2021). Very conspicuous bands are seen between approximately 2850 and 3000 cm^−1^, which shows the asymmetric and symmetric stretching of C‐H‐CH_2_. This band represents aliphatic compounds that are present in organic compounds such as fatty acids (Okoro, Sharifi, Jesson, Bridgeman, & Moruzzi, 2021). It can be seen that these bands were significantly reduced after the fractionation procedures. The oil in kenaf seed contains a high phospholipid content (Mariod et al., 2010; Mohamed et al., 1995), so its removal appears to have caused the reduction of the peaks. The presence of carboxylic acid and amide groups are very prominent in the CrKP, GloKP, and AlbKP while other KPFs recorded low transmittance of this band (1721–1550 cm^−1^). The presence of protein is confirmed by strong amide I and II bands, respectively, as seen in bands 1550 and 1560 cm^−1^. Other bands show the presence of compounds having coagulation abilities, such as phenol (1237 and 1243 cm^−1^) and polysaccharides (1015 and 800 cm^−1^).

**FIGURE 2:**
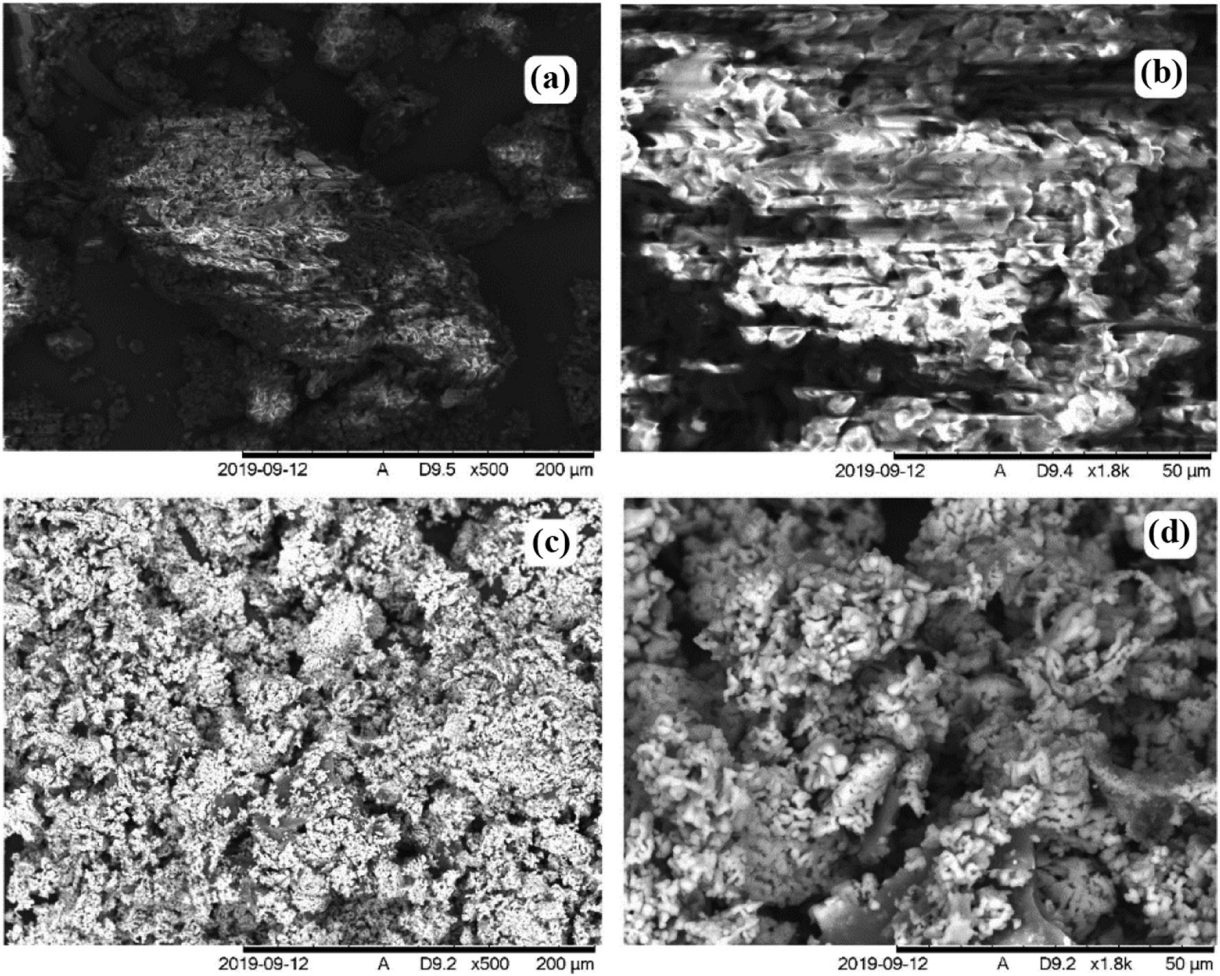
Scanning electron microscopy images of KCPs showing (a) an extreme close‐up of the CrKP and (b) a magnified view of (a), while (c) and (d) show the structure of GloKP after extraction at the same scales as (a) and (b), respectively.

Scanning electron microscopy (SEM) of flocs was undertaken to understand the surface morphology of the GloKP. The SEM images obtained for GloKP were compared with the CrKP for variations in sizes, structures, and appearance (Figure 2). Comparing GloKP with CrKP with a magnification of 500 and 1800, it was observed that the GloKP granules had smoother, solid surfaces with no visible pores compared with CrKP, which had flake‐like structures with granules joined together. The granule size of both CrKP and GloKP is predominantly between 10 and 50 μm, although for GloKP, there were clustered and cemented granules whose sizes could not be determined. The GloKP granules had an organized structure, which was mostly of spherical edges with no crystalline structures. These GloKP granules are hypothesized to have enhanced the flocculation performance partly because of the delipidation process (Gassenschmidt et al., 1995; Okoro, Sharifi, Jesson, & Bridgeman, 2021). In contrast, CrKP contains phospholipids that reduce the polymer's reactive surface and reduces interaction with colloidal particles in the untreated water (Okoro, Sharifi, Jesson, Bridgeman, & Moruzzi, 2021).

### Influence of kenaf protein fraction dosages on water turbidity

**FIGURE 3:**
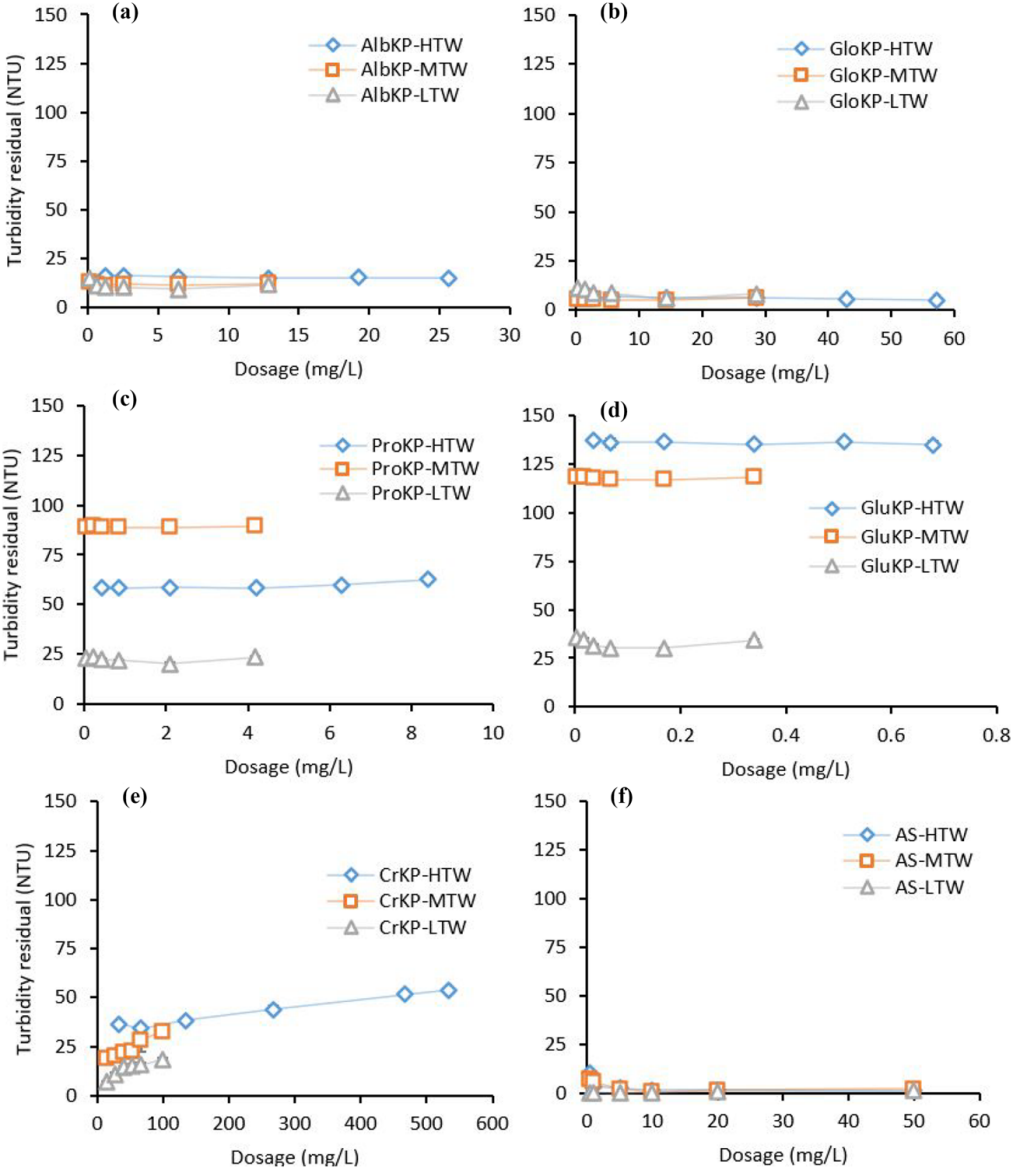
Selection of optimum dosage (protein concentration) for AlbKP, GloKP, ProKP, GluKP, CrKP, and the AS; turbidity values measured after 70 min; experimental condition: water turbidity (HTW‐510, MTW‐150; LTW‐32 NTU), pH = 7; T = 20°C. CrKP dosages are derived from optimum test conducted in Okoro, Sharifi, Jesson, Bridgeman, and Moruzzi (2021).

The required KPF dosages for the untreated water were determined by jar testing, with results shown in (Figure 3). For all water types, that is, HTW, MTW and LTW, and GluKP and ProKP (Figure 3c,d), showed inferior turbidity removal compared with the crude extract, CrKP. Consequently, these fractions are not discussed in detail in the remainder of this paper, although results are included in figures for comparison. Conversely, the performance of AlbKP and GloKP (Figure 3a,b) was better than CrKP, and variation in turbidity removal with dosage was evident.

Increasing AlbKP dosage from 1.3 L to 26 mg/L in HTW resulted in a decrease of residual turbidity (Figure 3a). From the figure, using AlbKP in MTW and LTW showed an initially decreasing then increasing trend. Similarly, GloKP use in both MTW and LTW showed the best turbidity removal rate when a dosage of 14 mg/L was used, and any dosage below and beyond this value increased turbidity. The results obtained for AlbKP indicate that the optimum coagulation–flocculation process in the MTW and LTW would be achieved when coagulants dosage is 6 mg/L, which is lower than the optimum dosage required in the HTW. An observation made during the use of GloKP in HTW is the continuous improvement in turbidity removal up to a dosage of 57 mg/L. Even though high dosages of GloKP resulted in improved turbidity removal in HTW, the high dosage increased the dissolved organic content of treated water. Further discussion on DOC is available in Section 3.3.

As previously stated, only the AlbKP and GloKP gave removal rates above the CrKP. During the CrKP experiments, a linear increase in residual turbidity was recorded for HTW (Figure 3) as CrKP dosage was increased, implying its poor suitability in water treatment. The improved performances of the AlbKP and GloKP are expected as both are refined forms of the CrKP that have undergone defatting and other purification procedures to eliminate non‐coagulating compounds. These non‐coagulating compounds, such as lipids, have been reported to form a barrier around the coagulant, thereby limiting their efficacy (Gassenschmidt et al., 1995). Only GloKP gave a comparable performance to that of AS and achieved turbidity values near the WHO specified maximum contaminant level (MCL) value of 4 NTU for point‐of‐use drinking water treatment (WHO, 2012).

**FIGURE 4:**
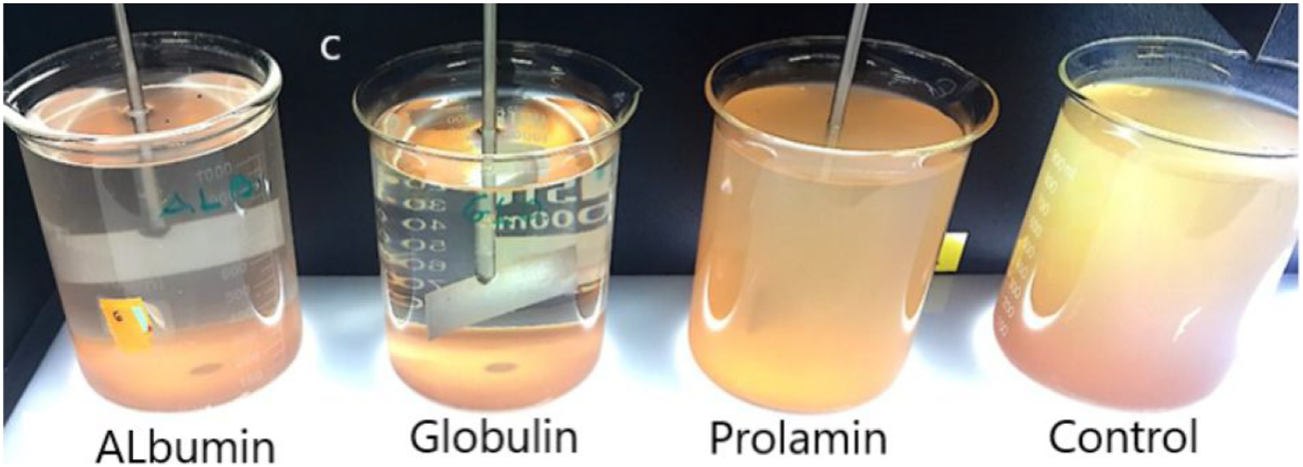
The effect of KPFs on the removal of NOM in MTW. Photographic evidence shown is for optimum dosages of AlbKP (6.42 mg/L), GloKP (14 mg/L), and ProKP (2.1 mg/L) and a blank uncoagulated having no KPF dosage. Experimental conditions: pH 7; T = 20°C. GluKP is not included because cloudiness was higher than all trials including control (untreated water), so it is omitted.

Water turbidity indicates the degree to which the transparency of water is lost due to suspended particles. Figure 4 shows photos taken after water treated with KPFs had settled for 70 min, illustrating the different level of turbidity. The degree of cloudiness indicates the concentration of particles that are individually invisible to the human eye. As can be seen, the residual particles decreased from the ProKP to GloKP, with the uncoagulated (blank) sample stable throughout the observation period. Leaving these beakers for an extended settling time of 24 h increased the clarity of the water and the particle removal. The visual performance reveals that KPFs have a different effect on turbid water. The result strongly confirms that GloKP is potentially a good performing coagulant with good particle destabilization ability. Also, it indicates that GloKP produced the optimum performance of the examined fractionates.

**FIGURE 5:**
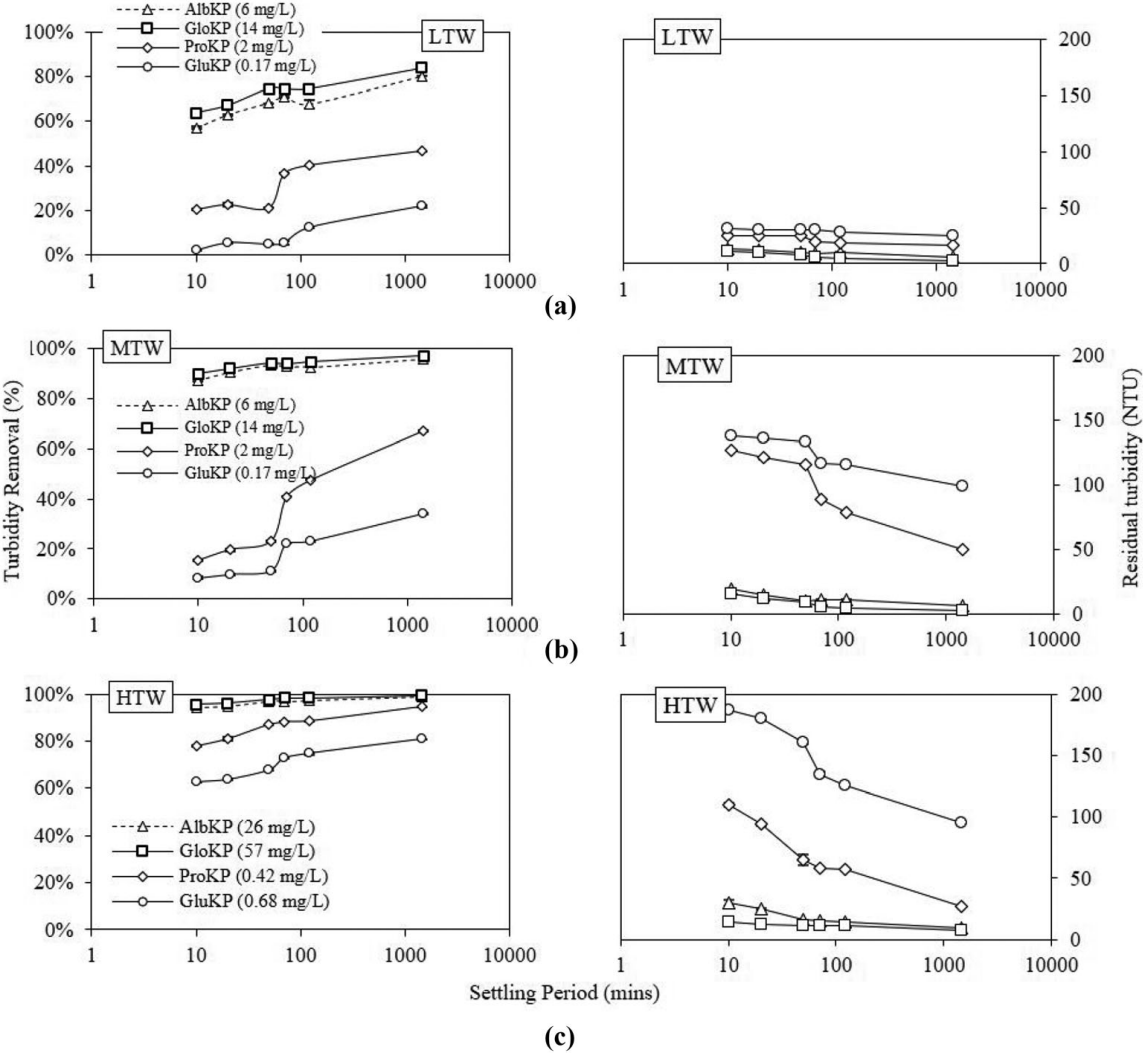
Turbidity residual‐settling time profile for KPFs in (a) LTW, (b) MTW, and (c) HTW. Experimental condition: pH = 7, T = 20°C

Total suspended solids concentration is an important factor used in observing water clarity and quality. The optimum dosages were used in studying the performance of KPFs over a 24‐h period. The residual turbidity of HTW with optimum dosages of AlKP and GloKP after 70‐min settling time were 15 and 5 NTU (Figure 5), and their corresponding removal rate exceeded the untreated water by 20% and 22%. The relatively high reduction seen for the uncoagulated sample was due to the high collision rate caused by high particle concentration in water, a phenomenon reported in earlier work using *Moringa oleifera* (MO) (Muyibi & Evison, 1995). For the MTW, optimum dosages used were 6.4 and 14 mg/L for the AlbKP and GloKP, respectively. AlbKP and GloKP had removed 92.5% and 96.6% of the turbidity, respectively, compared with 20% for the uncoagulated sample. With the same KPF dosages, RT values in LTW were 9 (AlbKP) and 6 NTU (GloKP), exceeding the uncoagulated sample removal rate (10%) by 61% and 71%, respectively.

Overall, the RT of all the KPFs reduced over the 24‐h period. This gradual and continuous reduction recorded is as a result of sedimentation brought by the increased particle‐coagulant interaction. It is possible that even after the slow stir phase, the protein and other coagulating compounds in the KPFs continued reacting, which resulted in continuous adsorption and binding action. After a 24‐h settling period, the residual turbidity recorded for the uncoagulated sample was 80 NTU, and the values recorded for treatment using AlbKP and GloKP in the HTW were 10 and 2 NTU, respectively, and 7 and 3 NTU in the MTW. For the LTW, the residual turbidity values obtained after the 24‐h period were 6 and 2 NTU. From the values obtained, the lowest RT was obtained when GloKP was used. Importantly, the RT with GloKP as coagulant was within the WHO maximum allowable limit for turbidity for all water types, signifying that with a 24‐h settling time potable water can be achieved using GloKP.

### Contribution of kenaf protein fraction to the organic matter load of treated water

**FIGURE 6:**
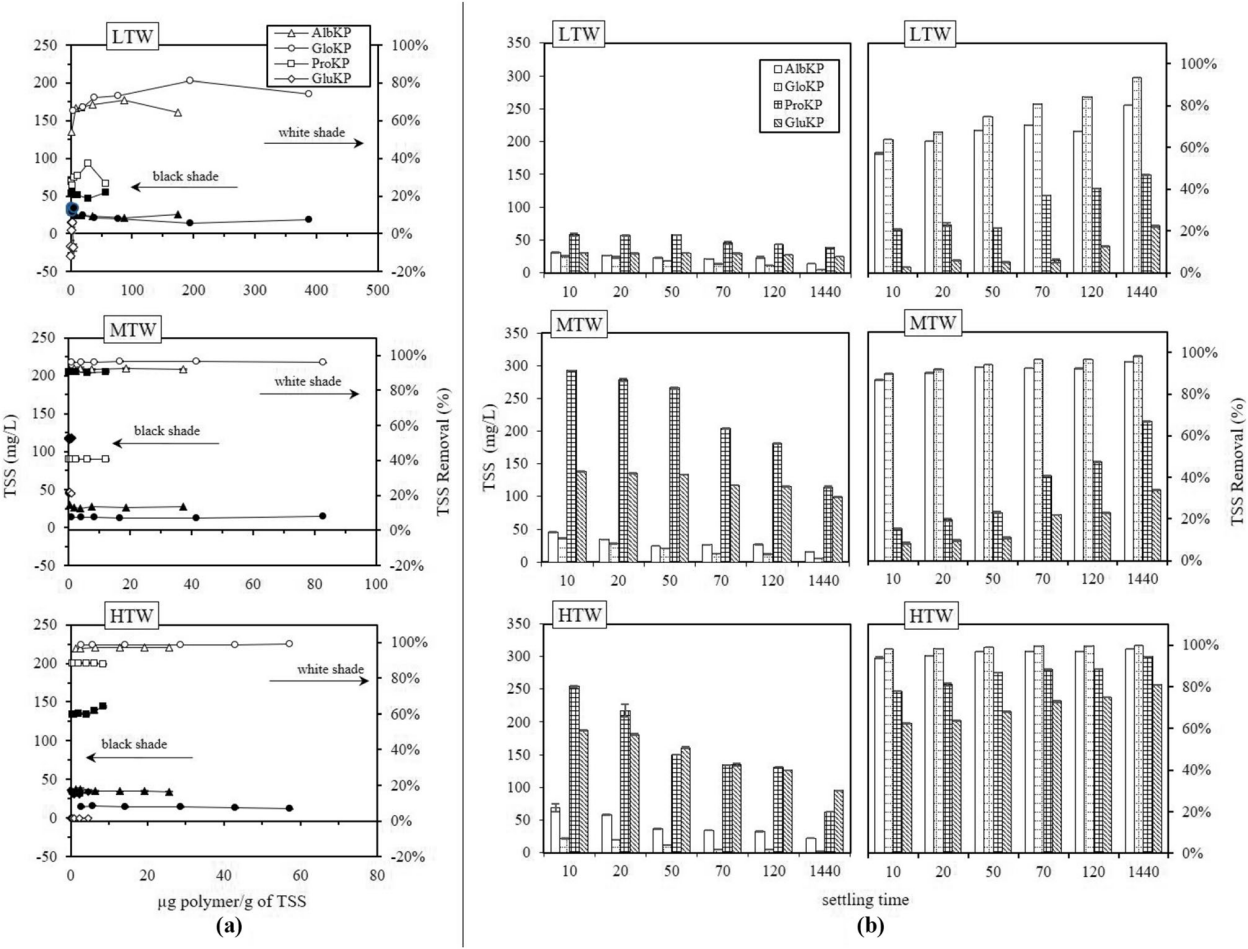
Effect of (a) different KPFs dosages on TSS (b) settling time on optimum KPFs dosage. Conditions: settling period = 70 min (for “a”), pH = 7

TSS concentration for the LTW, MTW, and HTW were approximately 74, 345, and 1173 mg/L, and optimum dosages for AlbKP and GloKP used in flocculating suspended particles in the LTW were 87 and 194 μg polymer/g TSS. In MTW, optimum dosages were 19 and 41 μg polymer/g TSS, while for HTW, the optimum dosages were 22 and 49 μg polymer/g TSS, respectively. Typical optimal dosages reported in the literature are approximately 1 mg/g of suspended solids or less when synthetic polymers are applied (Bolto & Gregory, 2007). KPFs concentration, as well as their chemical properties, affected their flocculation ability, as indicated in Figure 6. The quality of water produced by optimum AlbKP dosage of 87 μg/g in LTW was below quality obtained for the GloKP, as indicated in their corresponding TSS values of 22 and 6 mg/L. When the dosage increase passes the optimum value, the TSS ascends, and this results in high cloudiness of the water due to solids addition from the polymer and restabilization of floc.

Considering the TSS of the LTW, it is clear that the low collision rate influenced the TSS removal due to low impact and interaction of the suspended particles. A similar performance exists for the MTW and HTW with polymer requiring a higher optimum dosage than the LTW, which is typical of polymer bridging flocculation (Gregory, 2013). Increasing the AlbKP and GloKP polymer dosages up to their optimum values did not influence the TSS removal. However, polymer dosages above the optimum values increased the solids load of the treated water. At the optimum dosage, the KPFs adsorbs on the particles' surfaces forming linkages or chains with the aid of the collision forces. Excessive dosages caused a restabilization of particles because of oversaturation of attachment sites. More details are provided in Section 4. Figure 6b indicates the KPFs' performance under different retention time. TSS concentration at 70‐min residence time, varied from 14 to 82, 12 to 273, and 12 to 316 mg/L in the LTW, MTW, and HTW, respectively. The lowest TSS values were recorded for the optimum dosages, although no KPFs could entirely remove the TSS. An increase in the residence time generally resulted in lower TSS concentration due to the prolonged period for flocs to settle, and a similar mechanism of bridge flocculation must have played out.

**FIGURE 7:**
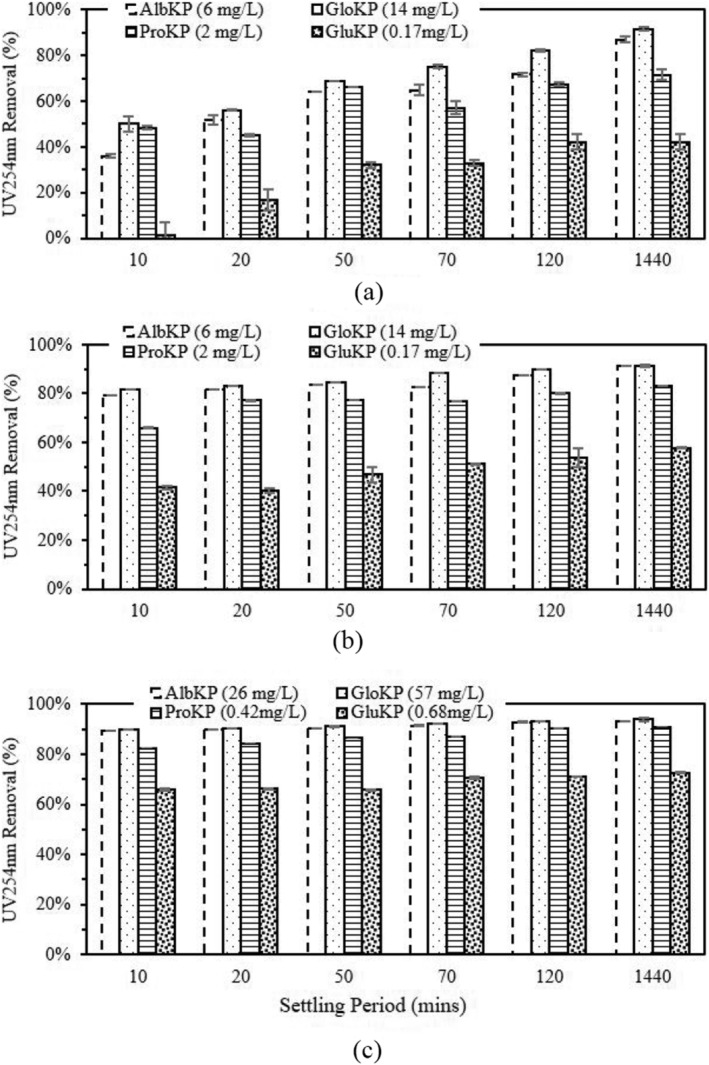
UV_254nm_ absorbance‐sedimentation profile for evaluating the performance of KPFs in (a) LTW, (b) MTW, and (c) HTW. Experimental condition: untreated water, pH = 7, T = 20°C. Vertical bars show experimental uncertainty.

One important organic matter indicator used for water quality monitoring is the ultraviolet absorbance at 254 nm. As shown in Figure 7, the UV_254_ removal percentage was generally high and similar to the turbidity removal. For the HTW and MTW, the highest UV_254_ removal after 70‐min settling period was obtained in GloKP treated water, with values of 92% and 89% for HTW and MTW, respectively. AlbKP improved the UV_254_ removal by 26% and 36% above the removals recorded for the uncoagulated samples, which were 65% and 47%, respectively. Evaluating KPFs performance in LTW (Figure 7b) revealed a similar trend to the HTW and MTW where the GloKP had the lowest UV_254_ value of 75% and was 35% above the uncoagulated sample percentage of 39%. After the 24‐h settling period, the UV_254_ removal value for AlbKP and GloKP, which was recorded for all water types, increased above 87%. KPFs with higher protein concentration gave the best removal rates indicating that the protein content in the extracts was key to the coagulation–flocculation process. Also, based on our previous work (Okoro, Sharifi, Jesson, Bridgeman, & Moruzzi, 2021), the solubilization process using salt (NaCl) improved the protein dissolution process, possibly leading to an improved polymer‐particle interaction.

**FIGURE 8:**
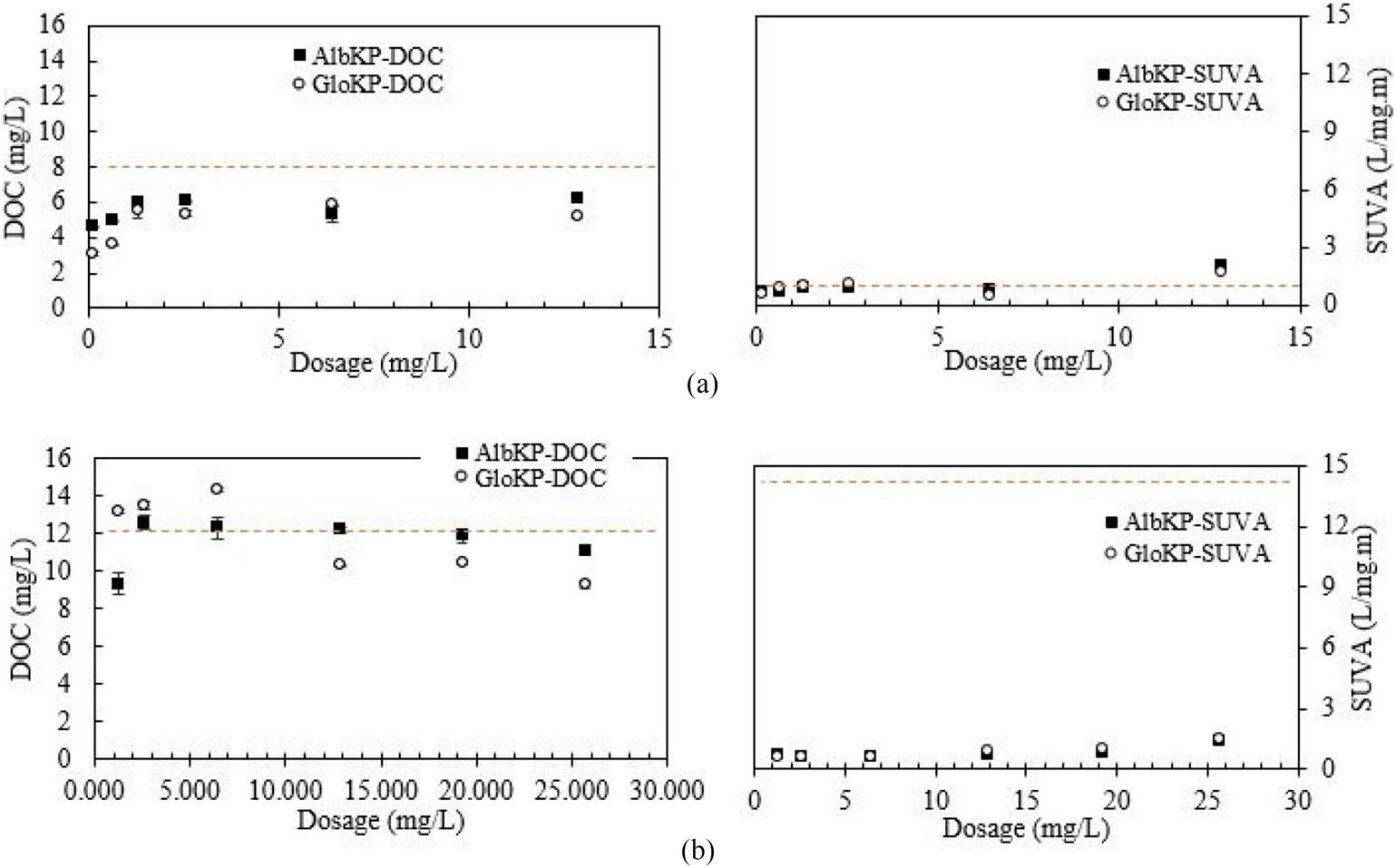
DOC and SUVA_254_ concentration obtained for KPFs in (a) LTW and (b) HTW. Experimental condition: untreated water, T = 20°C, pH for a and b = 7, SUVA_LTW_ = 1.7 L/mg‐m, SUVA _HTW_ = 14.8 mg/L, DOC_LTW_ = 8 mg/L, DOC_HTW_ = 12.1 mg/L. Control values in dotted

Another indicator used to assess the concentration and nature of the organic matter present in water is the SUVA_254_. The SUVA_254_ value examined in this work classified the treated water based on the nature of the organic matter present. The SUVA_254_ values obtained with different dosages of the KPFs in LTW are shown in Figure 8a. The SUVA_254_ values in this paper distinguished the NOM as hydrophobic, hydrophilic, or aromatic, which require a different dosage of polymers for effective flocculation. The figure reveals that increasing the dosage of AlbKP from 0.1 to 12 mg/L in LTW of DOC 8 mg/L caused the SUVA_254_ and DOC values to increase. The SUVA_254_ values for AlbKP and GloKP varies from 0.5 to 2.1 L/mg‐m, and GloKP values were slightly lower than the AlbKP. Compared with the untreated water SUVA_254_ value of 1.7 L/mg‐m, the GloKP optimum dosage produced the lowest value of 0.5 L/mg‐m. The SUVA_254_ values were approximately 2, which indicates a blend of hydrophobic humic and hydrophilic non‐humic substances (Matilainen et al., 2011). The increased SUVA_254_ values recorded with increasing dosage is expected because the GloKP is of plant origin and contains a large variety of organic compounds, which might have leached out and contributed to the SUVA_254_ noted in the study. The use of both AlbKP and GloKP is expected to exert a high chlorine demand during the disinfection process, potentially resulting in the formation of high concentrations of disinfection by‐products (Edzwald & Tobiason, 1999).

In the HTW, the SUVA_254_ value depicted in Figure 8b showed a wider range of NOM from 0.6 to 1.5 L/mg‐m. SUVA_254_ values across the AlbKP dosages yielded a near‐linear curve revealing the influence the increasing dosages had on the DOC removal. Comparing the DOC of the untreated water sample (12 mg/L) to the AlbKP performance evaluated at 70 min indicated a poor DOC removal rate across all dosages, that is, −4% to 23% removal rate, with the optimum AlbKP dosage giving DOC value of 11 mg/L. Likewise, the SUVA_254_ value for the GloKP improved as the DOC reduced with the UV_254_ correlating strongly (>90%) with the DOC in most cases. This trend was also observed for other KPFs used in this study. A strong correlation of the UV_254_ value with the DOC in humic‐rich water has been reported elsewhere in the literature (Chow et al., 2005). Water treated using GloKP had a maximum SUVA_254_ value of 1.5 L/mg‐m, which was a 90% reduction in the untreated water value of 14.8 L/mg‐m. SUVA_254_ values for GloKP in HTW were similar to those of the LTW. The value showed that the organic matter was composed of low mW hydrophilic non‐humic matter, which requires additional efforts like the enhanced coagulation for easier removal (Liang & Singer, 2003).

**FIGURE 9:**
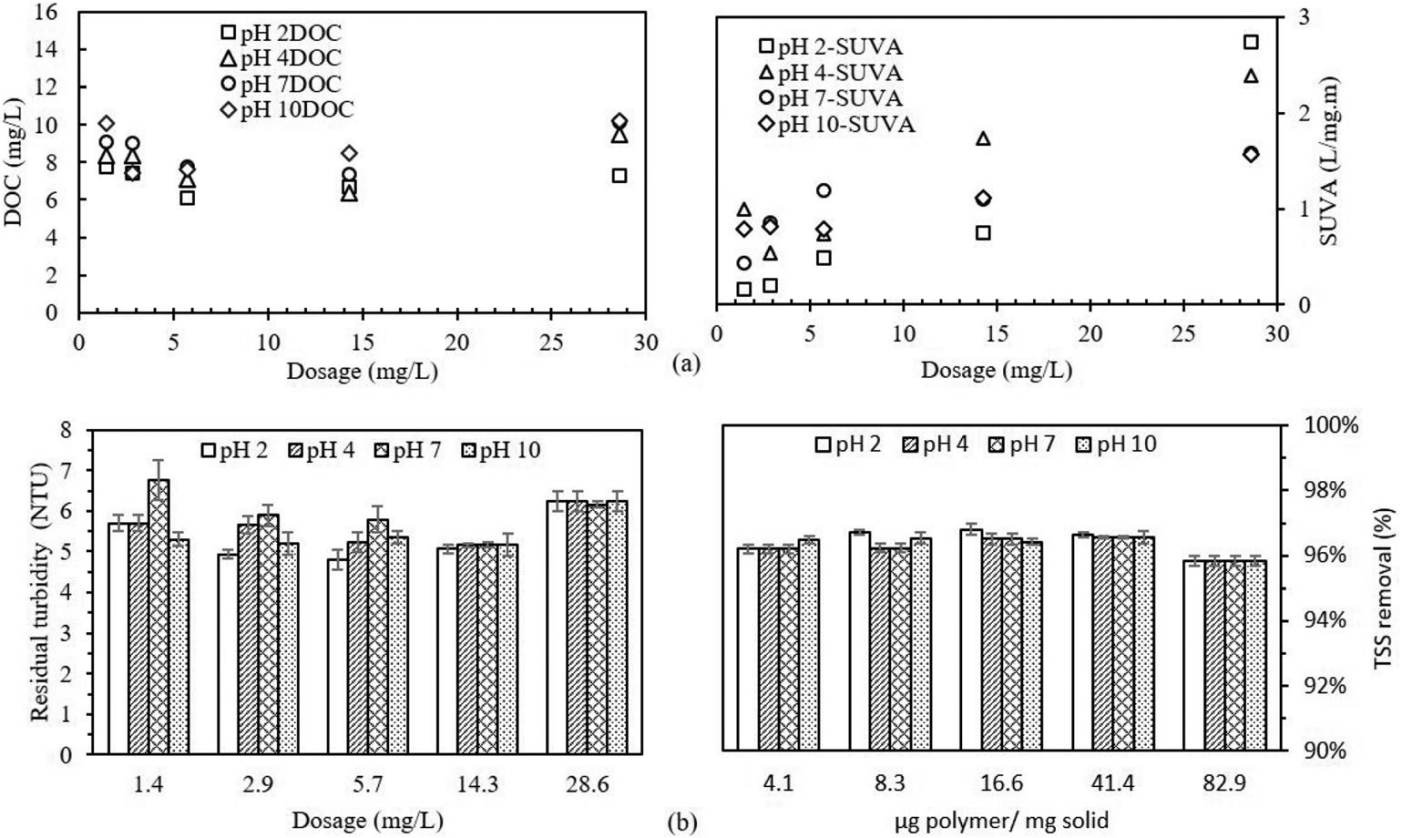
(a) SUVA_254_ and DOC values for improved coagulation using GloKP in MTW under an operating pH of 2, 4, and 10. (b) Selection of optimum dosage of GloKP under solution pH of 2, 4, and 10. Experimental condition: untreated water, T = 20°C. Vertical bars show experimental uncertainty. DOC_MTW_ = 10.2 mg/L, SUVA_MTW_ = 7.0 L/mg‐m, Turbidity_MTW_ = 150NTU, TSS_MTW_ = 345 mg/L

Further experiments conducted using KPFs in MTW and under different pH revealed that pH influences the removal of organic matter during water treatment using KPFs. The results for these are illustrated in Figure 9a and are for GloKP, the best performing KPF, under a range of dosages and pH. The two‐way ANOVA conducted showed a significant difference (*p* < 0.05) between the acidic and alkaline‐based GloKP treated water (F = 8.25, *p* = 0.01) under the same dosage and pH of 2, 4, 7, and 10. Similar DOC and SUVA trends, although not reported here, indicate a significant difference at higher dosages. Water at pH 2, as shown in Figure 9a, had a maximum SUVA_254_ value of 2.74 L/mg‐m. The DOC curve for the range of increasing dosages used was parabolic with the GloKP optimum dosage giving the lowest DOC of 6.1 mg/L. Water at pH 4, 7, and 10 also yielded a qualitatively similar pattern to the pH 2 sample, with optimum dosage giving SUVA_254_ values of 1.7, 1.1, and 1.1 L/mg‐m, respectively. SUVA_254_ values for pH 7 and 10 indicated that the NOM was predominantly of non‐humic hydrophilic materials while values for pH 2 and pH 4 showed that the NOM was made up of particles with both high and low molecular weight. All GloKP dosages across the different pH ranges gave residual turbidity values higher than the MCL of 4 NTU and TSS removal of approximately 96% as shown in coagulation optimization chart for the pH (Figure 9b), suggesting that their high concentration can limit treatment performance. Although pH 2 is where the treatment is most effective for turbidity removal, the removal rates remain high at other pHs.

**FIGURE 10:**
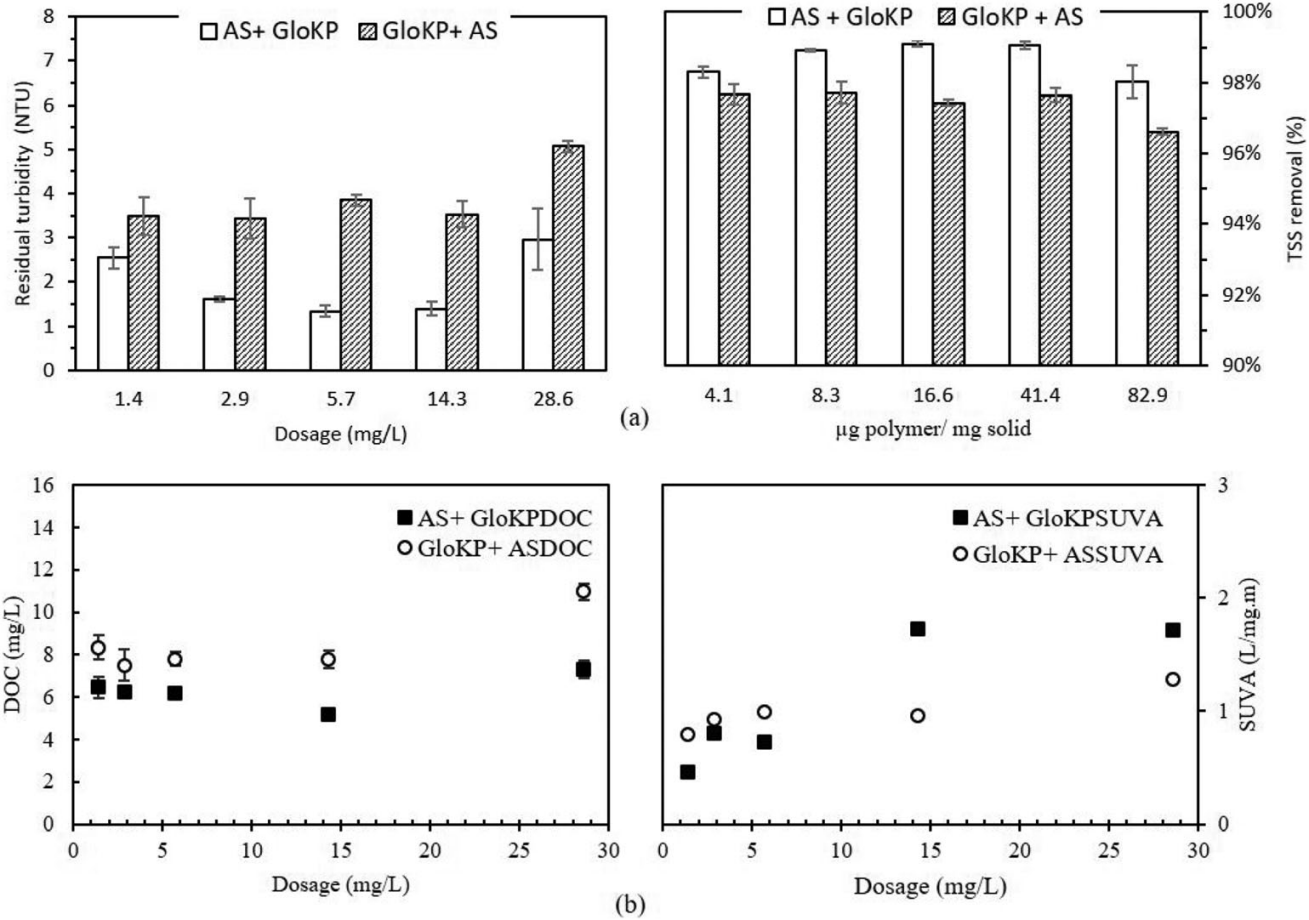
(a) Selection of optimum dosage for AS + GloKP and GloKP + AS in MTW; turbidity values and TSS were measured after 70 min (b) DOC and SUVA_254_ values when dual coagulants AS + GloKP and GloKP + AS were used in MTW. Experimental condition: untreated water, T = 20°C. Vertical bars show experimental uncertainty. Turbidity_MTW_ = 150 NTU, TSS_MTW_ = 345 mg/L, DOC_MTW_ = 10.2 mg/L, SUVA_MTW_ = 7.0 L/mg‐m, AS = 5 mg/L

As previously shown in Figure 3f, the use of AS (especially the optimum dosage) resulted in a rapid reduction of the residual water turbidity. However, in order to leverage the benefits of PCPs, it is potentially beneficial to use a combination of AS with KPFs. At the time of this report, the DOC values for AS experiment were not available due to technical challenges. However, evidence of the benefit of combining AS with the PCPs are illustrated in the supplementary figure provided (Figure S1). Optimization tests conducted on the dual coagulants (GloKP + AS and AS + GloKP, where in the former GloKP acted as a primary coagulant and the latter as the coagulant aid) determined the best dosage for the removal of NOM and turbidity from the water. Analyzing the curve of the dual coagulants (Figure 10a) reveals a parabolic curve with the minimum and maximum RT values of 1.3 and 3.0 NTU when GloKP was used as the aid, at dosages of 5.7 and 29 mg/L, respectively. A higher RT value was obtained when GloKP was used as the primary coagulant. The highest and lowest RT values recorded were 3.5 and 5.1 NTU for GloKP dosage of 2.9 and 29 mg/L, respectively, with negligible variation across the range up to 15 mg/L. Similarly, from Figure 10a, the lowest TSS removal occurred when GloKP was used as a primary coagulant indicating the addition of organic materials from the polymeric compound.

For the coagulants and coagulant aid combination shown in Figure 10b, the SUVA_254_ values obtained showed similar trends with the maximum value (1.7 L/mg‐m) recorded when 14 mg/L of GloKP was used as a coagulant aid. For both dual coagulants, the SUVA_254_ data indicate that organic matter in the water was predominantly of non‐humic origin, which includes materials that are released during the coagulation–flocculation process originating from plant cells such as protein, lipids, sugar, amino acids, and starch. This is likely to be due to the GloKP being purely of plant origin and containing a large variety of these compounds, and these leaching out to contribute to the SUVA_254_ noted during the study. A similar observation was made of the DOC value.

## FLOCCULATION EVIDENCE FROM EM AND ZETA POTENTIAL OF SUSPENDED PARTICLES

**FIGURE 11:**
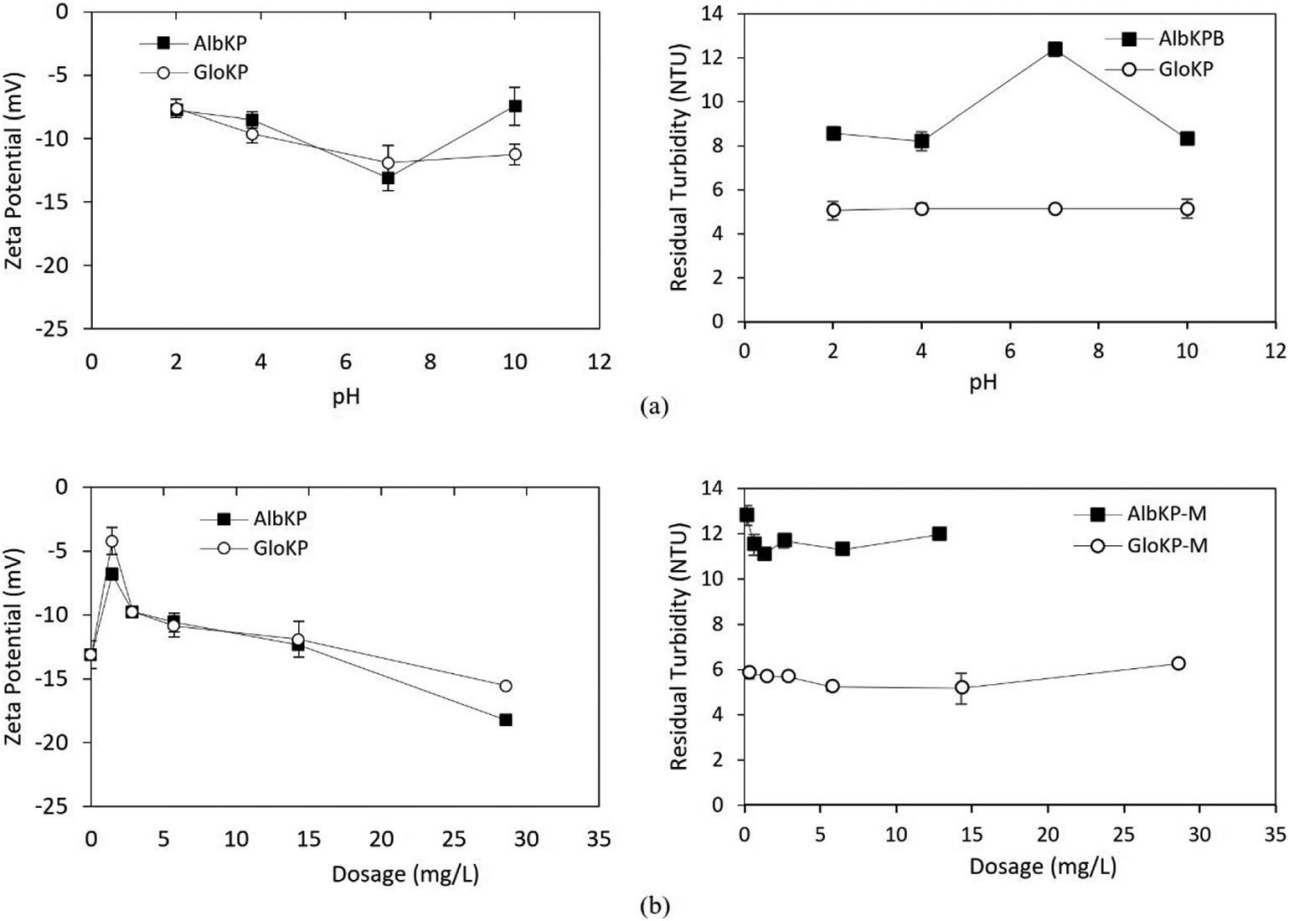
(a) Zeta potential and residual turbidity—pH profile of AlbKP and GloKP in MTW using optimum dosages. (b) Zeta potential and residual turbidity—dosages profile of AlbKP and GloKP in MTW. Conditions: settling period = 70 min; error bars signify experimental uncertainty.

The relationship between the zeta potential, pH, and RT value is shown in Figure 11a. Two‐way ANOVA showed that the pH value significantly (*p* < 0.05) affects the zeta potential and the RT value obtained. It is also clear that the poorly performing KCFs dosages (Figure 11b) had significantly higher zeta potential magnitudes (*F* = 77.89, *p* = 0.00). Variation of RT with pH is also statistically significant (*F* = 7.15, *p* = 0.01). Similarly, a significant difference existed across the RT values studied using the different KPFs (*F* = 73.55, *p* = 0.00). The AS used in this study was at approximately pH 6.8, which was within the range reported by (Zhao et al., 2009). From the figure and results obtained, the zeta potential of AS was 4.74 mV and was of a lower magnitude than that of the AlbKP, GloKP, and both the dual coagulants used.

**FIGURE 12:**
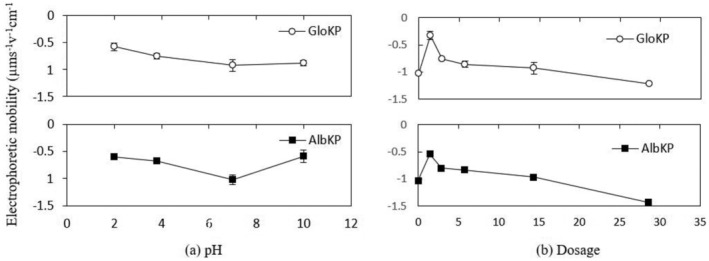
(a) Electrophoretic mobility‐pH profile of AlbKP and GloKP in MTW. (b) Electrophoretic mobility‐dosage profile AlbKP and GloKP in MTW at pH 7. Conditions: settling period = 70 min; T = 20°C; error bars signify experimental uncertainty.

**FIGURE 13:**
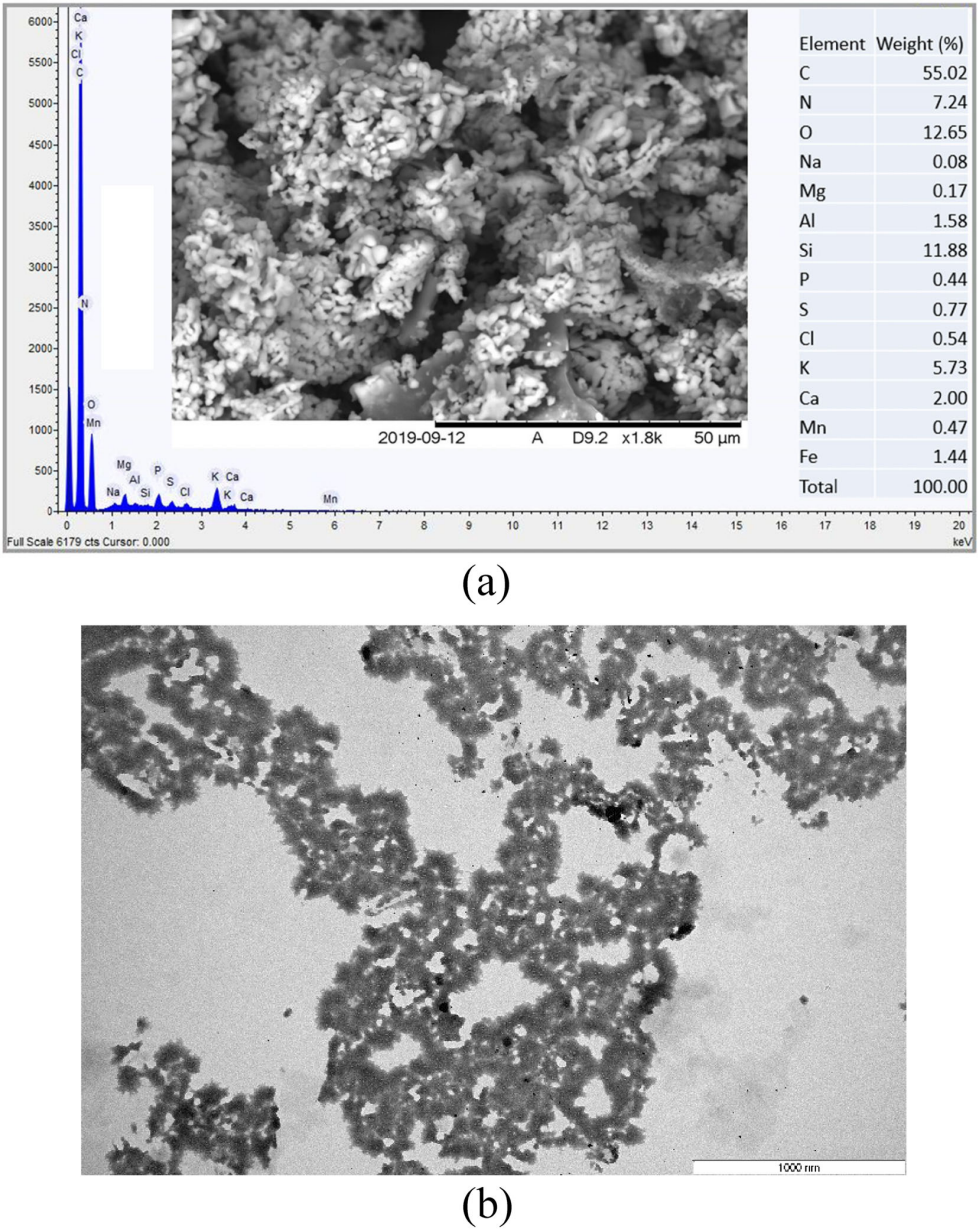
(a) Scanning electron microscopy image of GloKP before water treatment, revealing the EDAX elemental analysis. (b) Transmission electron microscopy close‐up images of flocs produced, showing the possible polymer‐particle interaction through adsorption bridging

From Figure 11a, the zeta potential of AlbKP and GloKP dropped to a minimum at pH 7 and then recovered as the pH moved from the acidic to the alkaline region. However, a reduction in the zeta potential is observed mainly for AlbKP at the neutral pH, indicating possible deprotonation of the acidic functional groups such as the phenolic and carboxylic, increasing anionic sites leading to higher particle restabilization. The low repulsion recorded is confirmed by the particle mobility of −1.02 and −0.93 μm s^−1^v^−1^ cm^−1^, for the optimum dosage of AlbKP and GloKP, respectively (Figure 12a). At neutral pH, increasing their dosages reduced the particles' positive mobility (Figure 12b) and zeta potential, thereby resulting in a rise of the RT value due to a net repulsion between particles and polymer charge. It is clear from the particles' mobility pattern that increasing the coagulant dosage from 1.4 to 29 mg/L resulted in the addition of more anions in solution. Taking into account the particle mobility pattern and RT curve, it can be concluded that neither charge neutralization nor sweep flocculation was the dominant mechanism. In fact, the EM of the AlKP and GloKP‐particle complexes remained below zero for all pHs and dosages confirming that the polymer was unable to neutralize the particles in suspension. The polymer‐particle morphology and complexes (Figure 13a,b) indicate that the particles' rearrangement resembles one of adsorption bridging with polymer‐like chains visibly adsorbed on the oppositely charged particles. Therefore, polymer bridging, enabled by the adsorption of the KPFs onto the surface of the particles during particle collisions, is the likely cause of the floc formation.

Variation in performance of GloKP is typical of the bridging flocculation mechanism. The mobility pattern indicated in Figure 12 is conceivably the result of surface complexation caused by ligands with opposite charges (Miller et al., 2008). Formation of complexes can occur between the anionic KPFs and organic matter. The complex ions formed involved more than one chemical species having an overall charge. The metal cations present in these polymeric compounds (Figure 13a) can readily bind to existing charged sites to form complexes depending on their concentration and the solution pH. Considering the zero point charge (IEP) for organic matter and GloKP gave approximately 3.5 (Miller et al., 2008) and approximately 1.5 (Okoro, Sharifi, Jesson, Bridgeman, & Moruzzi, 2021), respectively, and, below this charge, the adsorbent surface was protonated. It is envisaged that at neutral pH, both the NOM and KPFs, would only exhibit a net negative surface charge. Moreover, the GloKP performance decreases at dosages away from the optimum value, and this behavior was previously reported to be typical with the bridging mechanism (Oladoja, 2015). Also, FT‐IR spectrum (Figure 1) revealed that the KPFs surface had a variable distribution of coagulating compounds such as amino (–NH_2_), carboxylic (R‐COOH), and phenolic groups (–OH), and this matches findings in the literature for other PCPs (Yazan et al., 2011). A study using mustard oil cake (MOC) reported that at acidic pH, amine was in the protonated form (–NH_3_^+^) and adsorbed oxyanions through an ion‐exchange mechanism (Reddy et al., 2017). The phenolic groups present in the kenaf seed reveals that it is anionic and can easily deprotonate to form phenoxide (Yin, 2010). Another study also revealed that the carboxylic group and lignin groups present on the surface of MOC could behave as an electron donor (Krishnani et al., 2008). These compounds, which were also found in KPFs, can enhance the electrostatic attraction necessary for the bridging action (La Mer, 1966).

**FIGURE 14:**
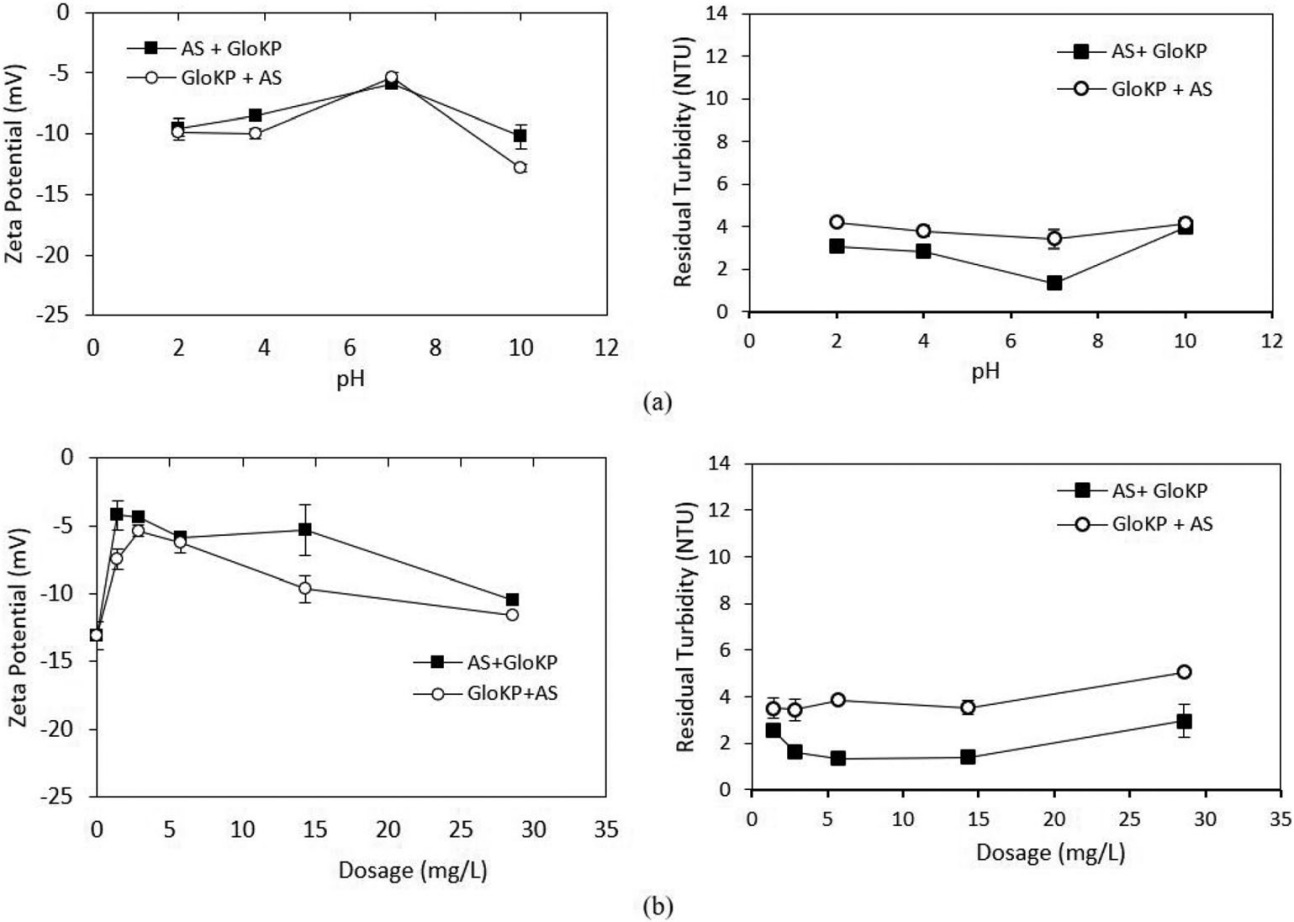
(a) Zeta potential and residual turbidity‐pH profile of dual coagulants in MTW using their optimum dosages. (b) Zeta potential and residual turbidity‐dosages profile of dual coagulants in MTW. Conditions: settling period = 70 min; error bars signify experimental uncertainty.

**FIGURE 15:**
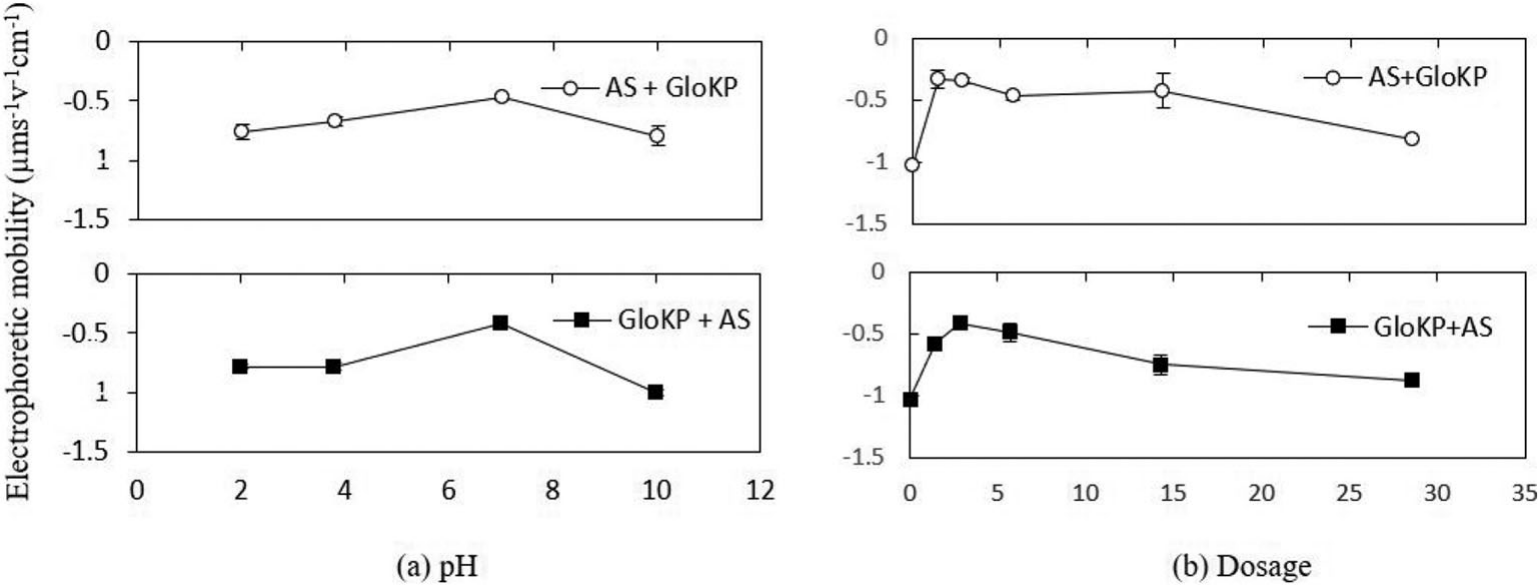
(a) Electrophoretic mobility‐pH profile of dual coagulants in MTW. (b) Electrophoretic mobility‐dosage profile of dual coagulants in MTW at pH 7. Conditions: settling period = 70 min; T = 20°C; error bars signify experimental uncertainty.

Depending on the operating conditions such as coagulant dosage and pH, AS can change the zeta potential of water to many different values (Black & Chen, 1965; Guo et al., 2015; He et al., 2019; Hu et al., 2012). The primary coagulation mechanism of AS is particle charge neutralization, which is facilitated by adsorbed precipitation (Liu et al., 2013) due to the alkaline condition of the test water. At acidic pH, GloKP slightly improves the attraction of negatively charged ions to their protonated surfaces (Figures 14a and 15a). Similar treatment conducted in an alkaline solution favors attraction of positively charged ions to the negatively charged surfaces (Crittenden et al., 2012). At neutral pH, the optimum dosage of the GloKP coagulant aid yielded a zeta potential value (−5.89 mV) above that obtained by GloKP alone (−12 mV) indicating the destabilizing influence of the AS. This increase was because of the hydrolysis of the monomeric and dimeric species of AS (Guo et al., 2015; Zhao et al., 2009), and the dominant coagulation mechanism experienced at this pH is likely to be a combination of charge neutralization by the AS, together with the adsorption and particle bridging action brought by the addition of GloKP. As the GloKP dosage is increased, the addition of more anion compounds occurs, thus adding more negative charged particles, which are reflected in the zeta potential recorded in Figure 14b. This is also confirmed by the negative particle mobility observed with increase in dosages of the coagulant aid (Figure 15b).

## CONCLUSION

In this study, the organic matter removal performance of KPFs was examined in low‐, medium‐, and high‐turbidity water (30, 150, and 500 NTU, respectively). Fourier transform infrared (FT‐IR) technique was used to examine the chemical fingerprint of the coagulants. Scanning electron microscopy coupled to dispersive energy analysis of X‐rays (SEM–EDAX) revealed the structure of the CrKP and GloKP surfaces. SUVA_254_, DOC, and turbidity values for a different range of KPFs dosages facilitated the monitoring of the organic matter concentration in both untreated and treated water. At the same time, the zeta potential and electrophoretic mobility values indicated the stability of the polymer‐particle suspension and also gave insight into the mobility of the suspended particles. The FT‐IR spectrum indicated that the KPFs surface have a different distribution of polymeric compounds such as amino (‐NH_2_), carboxylic (R‐COOH), and phenolic groups –OH groups, most of which can enhance the attraction required for particle destabilization. The results of this study revealed that the protein concentration was highest in GloKP, followed by AlbKP. The improved performance of GloKP was attributed to the salting‐in effect, which assisted in dissolving the active coagulation components. SUVA_254_ values suggest that the organic matter in GloKP is comprised mainly of non‐humic hydrophilic substances. Residual turbidity, UV_254_, SUVA_254_, and DOC values showed that the ProKP and GluKP poorly coagulated particulates in all the untreated water due to their organic matter content, which are of high molecular weight (mW) hydrophobic substances. The result shows that optimizing KPFs and dual coagulant dosage is crucial for maximum particle removal during water treatment.

When GloKP was used alone, the organic matter removal varied across the observed pH, with pH 2 exhibiting the highest removal efficiency, and the performance of GloKP decreased from the acidic to basic pH. Using both dual coagulants (GloKP and AS) gave DOC removal higher than GloKP alone. The results indicate that GloKP is a good alternative to its crude (CrKP) and solvent extract (HxKP) form. Based on the result of EM, it can be concluded that the primary coagulation mechanism for KPFs, especially GloKP, was adsorption‐bridging flocculation while AS was by particle charge neutralization. The dual coagulants benefitted from the combined influence of the two mechanisms resulting in an improved flocculation performance as indicated in floc properties.

This study has shown that GloKP can be used effectively both as a primary coagulant and as a coagulant aid in water treatment. Notwithstanding results from the UV_254_, SUVA_254_, and DOC, results indicate that GloKP can contribute to the organic matter load of treated water, making them potential precursors to disinfectant by‐product formation.

## AUTHOR CONTRIBUTIONS

**Benjamin U. Okoro:** Conceptualization; methodology; software; formal analysis; investigation; data curation; writing‐original draft. **Soroosh Sharifi:** Conceptualization; supervision; writing‐review and editing. **Mike Jesson:** Conceptualization; supervision; writing‐review and editing. **John Bridgeman:** Conceptualization; writing‐review and editing.

## Supporting information


**Table S1** Preliminary protein concentration of AlbKP and ProKP used for optimal dosage selection during coagulation‐flocculation experiments
**Table S2** Preliminary protein concentration of GloKP and GluKP used for optimal dosage selection during coagulation‐flocculation experiments
**Table S3** Preliminary protein concentration of CrKP used for optimal dosage selection during coagulation‐flocculation experiments
**Table S4** aluminium sulfate dosages used during coagulation‐flocculation experiments
**Figure S1** (a) floc growth rate of optimum dosages of HxKP, CrKP, AS and for different pH of GloKP. (b) the growth rate of AS, GloKP, GlobKP + AS and AS + GloKP after slow stir phase of 15mins. Operating conditions: turbidity (150 NTU), T = 20°C.

## Data Availability

The data that support the findings of this study are available on request from the corresponding author. The data are not publicly available due to privacy or ethical restrictions.
