# Supplementary material for: Protein fractionation of Hibiscus cannabinus (kenaf) seeds, its characterization, and potential use for water treatment
Source: Water Environ Res. 2022 Nov 12;94(11):e10805. doi: 10.1002/wer.10805 (PMC9828421; doi:10.1002/wer.10805)
Supplement: Supplementary file 1 — Table S1 Preliminary protein concentration of AlbKP and ProKP used for optimal dosage selection during coagulation‐flocculation experiments Table S2 Preliminary protein concentration of GloKP and GluKP used for optimal dosage selection during coagulation‐flocculation experiments Table S3 Preliminary protein concentration of CrKP used for optimal dosage selection during coagulation‐flocculation experiments Table S4 aluminium sulfate dosages used during coagulation‐flocculation experiments Figure S1 (a) floc growth rate of optimum dosages of HxKP, CrKP, AS and for different pH of GloKP. (b) the growth rate of AS, GloKP, GlobKP + AS and AS + GloKP after slow stir phase of 15mins. Operating conditions: turbidity (150 NTU), T = 20°C. [file WER-94-0-s001.docx]

Supplementary Tables and Figure

Title:

Protein fractionation of *Hibiscus cannabinus* (Kenaf) seeds, its characterization, and potential use for water treatment

Authors:

Benjamin U. Okoro^1^, Soroosh Sharifi^1^, Mike Jesson^1^, John Bridgeman^2^

Abbreviations: Albumin Kenaf product (AlbKP); aluminium sulphate (AS); Crude extracted products (CrKP); dV: Equivalent dosing volume; dWt: Equivalent dosing weight; High turbidity water (HTW); Low turbidity water (LTW); Medium turbidity water (MTW)

Table S1 Preliminary protein concentration of AlbKP and ProKP used for optimal dosage selection during coagulation-flocculation experiments

| AlbKP-HTW | | | | AlbKP-MTW | | | | | | | AlbKP-LTW | | | | | | |
| --- | --- | --- | --- | --- | --- | --- | --- | --- | --- | --- | --- | --- | --- | --- | --- | --- | --- |
| dV (mL/L) | Protein content (mg/L) | Mean (n=3) | SD | dV (mL/L) | Protein content (mg/L) | | Mean (n=3) | | SD | | dV (mL/L) | Protein content (mg/L) | | Mean (n=3) | | | SD |
| 1.0 | 1.3 | 16 | 0.14 | 0.1 | 0.13 | | 13 | | 0.35 | | 0.1 | 0.13 | | 15 | | | 0.61 |
| 2.0 | 2.6 | 16 | 0.55 | 0.5 | 0.64 | | 12 | | 0.50 | | 0.5 | 0.64 | | 11 | | | 0.52 |
| 5.0 | 6.4 | 15 | 0.30 | 1.0 | 1.3 | | 11 | | 0.98 | | 1.0 | 1.3 | | 11 | | | 0.48 |
| 10 | 13 | 15 | 0.06 | 2.0 | 2.6 | | 12 | | 0.35 | | 2.0 | 2.6 | | 10 | | | 0.24 |
| 15 | 19 | 15 | 0.21 | 5.0 | 6.4 | | 11 | | 0.13 | | 5.0 | 6.4 | | 9.3 | | | 0.08 |
| 20 | 26 | 15 | 0.13 | 10 | 13 | | 12 | | 0.17 | | 10 | 13 | | 11 | | | 0.32 |
| 25 | 32 | 15 | 0.14 | 15 | 19 | | 13 | | 0.53 | | 15 | 19 | | 12 | | | 0.34 |
| 30 | 39 | 17 | 0.21 | 20 | 26 | | 15 | | 0.09 | | 20 | 26 | | 14 | | | 0.17 |
| 35 | 45 | 21 | 1.53 | 25 | 32 | | 21 | | 1.70 | | 25 | 32 | | 16 | | | 0.09 |
| ProKP-HTW | | | | ProKP-MTW | | | | | | | ProKP-LTW | | | | | | |
| dV (mL/L) | Protein content (mg/L) | Mean (n=3) | SD | dV (mL/L) | | Protein content (mg/L) | | Mean (n=3) | | SD | dV (mL/L) | | Protein content (mg/L) | | Mean (n=3) | SD | |
| 1.0 | 0.4 | 58 | 0.93 | 0.1 | | 0.04 | | 89 | | 0.96 | 0.1 | | 0.04 | | 23 | 1.57 | |
| 2.0 | 0.8 | 58 | 0.55 | 0.5 | | 0.21 | | 89 | | 0.58 | 0.5 | | 0.21 | | 24 | 0.29 | |
| 5.0 | 2.1 | 59 | 0.32 | 1.0 | | 0.42 | | 89 | | 0.31 | 1 | | 0.42 | | 23 | 0.50 | |
| 10 | 4.2 | 58 | 0.19 | 2.0 | | 0.83 | | 89 | | 1.00 | 2 | | 0.83 | | 22 | 0.29 | |
| 15 | 6.3 | 60 | 1.05 | 5.0 | | 2.08 | | 89 | | 0.32 | 5 | | 2.1 | | 20 | 0.79 | |
| 20 | 8.4 | 63 | 0.55 | 10 | | 4.16 | | 89 | | 0.58 | 10 | | 4.2 | | 24 | 0.51 | |
| 25 | 11 | 65 | 1.42 | 15 | | 6.24 | | 90 | | 0.44 | 15 | | 6.2 | | 24 | 0.50 | |
| 30 | 13 | 68 | 0.68 | 20 | | 8.32 | | 91 | | 1.00 | 20 | | 8.3 | | 25 | 0.40 | |
| 35 | 15 | 75 | 1.44 | 25 | | 10 | | 93 | | 0.62 | 25 | | 10 | | 26 | 0.46 | |

**dV: equivalent dosing volume measured into the beakers in mL/L; SD: standard deviation**

Table S2 Preliminary protein concentration of GloKP and GluKP used for optimal dosage selection during coagulation-flocculation experiments

| GloKP-HTW | | | | GloKP-MTW | | | | GloKP-LTW | | | | | | | |
| --- | --- | --- | --- | --- | --- | --- | --- | --- | --- | --- | --- | --- | --- | --- | --- |
| dV (mL/L) | Protein content (mg/L) | Mean (n=3) | SD | dV (mL/L) | Protein content (mg/L) | Mean (n=3) | SD | | dV (mL/L) | Protein content (mg/L) | | Mean (n=3) | | SD | |
| 1.0 | 2.9 | 6.2 | 0.11 | 0.1 | 0.3 | 5.8 | 0.29 | | 0.1 | 0.3 | | 11 | | 0.84 | |
| 2.0 | 5.7 | 6.9 | 0.12 | 0.5 | 1.4 | 5.7 | 0.20 | | 0.5 | 1.4 | | 11 | | 0.40 | |
| 5.0 | 14 | 6.3 | 0.25 | 1.0 | 2.9 | 5.7 | 0.21 | | 1.0 | 2.9 | | 8.9 | | 0.20 | |
| 10 | 29 | 6.4 | 0.39 | 2.0 | 5.7 | 5.2 | 0.25 | | 2.0 | 5.7 | | 8.5 | | 0.50 | |
| 15 | 43 | 5.7 | 0.20 | 5.0 | 14 | 5.2 | 0.05 | | 5.0 | 14 | | 6.1 | | 0.11 | |
| 20 | 57 | 5.1 | 0.11 | 10 | 29 | 6.2 | 0.25 | | 10 | 29 | | 8.2 | | 0.12 | |
| 25 | 72 | 5.7 | 0.39 | 15 | 43 | 6.8 | 0.24 | | 15 | 43 | | 8.9 | | 0.06 | |
| 30 | 86 | 9.0 | 0.06 | 20 | 57 | 7.7 | 0.26 | | 20 | 57 | | 9.1 | | 0.04 | |
| 35 | 100 | 10 | 0.16 | 25 | 71 | 9.0 | 0.14 | | 25 | 71 | | 10 | | 0.12 | |
| GluKP-HTW | | | | GluKP-MTW | | | | | GluKP-LTW | | | | | | |
| dV (mL/L) | Protein content (mg/L) | Mean (n=3) | SD | dV (mL/L) | Protein content (mg/L) | Mean (n=3) | SD | | dV (mL/L) | | Protein content (mg/L) | | Mean (n=3) | | SD |
| 1.0 | 0.034 | 137 | 0.47 | 0.1 | 0.003 | 118 | 0.30 | | 0.1 | | 0.003 | | 36 | | 0.49 |
| 2.0 | 0.068 | 136 | 2.04 | 0.5 | 0.017 | 118 | 0.58 | | 0.5 | | 0.017 | | 34 | | 1.04 |
| 5.0 | 0.170 | 136 | 1.22 | 1.0 | 0.034 | 118 | 0.72 | | 1.0 | | 0.034 | | 31 | | 0.53 |
| 10 | 0.340 | 135 | 1.04 | 2.0 | 0.068 | 117 | 0.21 | | 2.0 | | 0.068 | | 30 | | 0.33 |
| 15 | 0.510 | 137 | 1.53 | 5.0 | 0.170 | 117 | 0.02 | | 5.0 | | 0.170 | | 30 | | 0.21 |
| 20 | 0.680 | 135 | 1.95 | 10 | 0.339 | 118 | 0.64 | | 10 | | 0.339 | | 34 | | 0.61 |
| 25 | 0.850 | 136 | 1.21 | 15 | 0.509 | 120 | 0.20 | | 15 | | 0.509 | | 35 | | 0.06 |
| 30 | 1.020 | 140 | 1.15 | 20 | 0.678 | 122 | 0.08 | | 20 | | 0.678 | | 40 | | 0.35 |
| 35 | 1.190 | 142 | 1.01 | 25 | 0.848 | 126 | 0.17 | | 25 | | 0.848 | | 47 | | 0.53 |

**dV: equivalent dosing volume measured into the beakers in mL/L; SD: standard deviation**

Table S3 Preliminary protein concentration of CrKP used for optimal dosage selection during coagulation-flocculation experiments

| CrKP-HTW | | | | CrKP-MTW | | | | CrKP-LTW | | | |
| --- | --- | --- | --- | --- | --- | --- | --- | --- | --- | --- | --- |
| dWt (mg/L) | Protein content (mg/L) | Mean (n=3) | SD | dWt (mg/L) | Protein content (mg/L) | Mean (n=3) | SD | dWt (mg/L) | Protein content (mg/L) | Mean (n=3) | SD |
| 50 | 33 | 36 | 0.51 | 20 | 13 | 19 | 0.10 | 20 | 13 | 7 | 0.08 |
| 100 | 67 | 34 | 0.26 | 40 | 27 | 20 | 1.79 | 40 | 27 | 11 | 0.90 |
| 200 | 133 | 38 | 1.42 | 60 | 40 | 22 | 2.59 | 60 | 40 | 14 | 0.50 |
| 400 | 267 | 44 | 2.00 | 80 | 53 | 23 | 3.21 | 80 | 53 | 15 | 0.91 |
| 700 | 467 | 52 | 1.50 | 100 | 67 | 28 | 5.69 | 100 | 67 | 16 | 1.08 |
| 800 | 534 | 54 | 1.00 | 150 | 100 | 33 | 3.06 | 150 | 100 | 18 | 0.65 |

**dWt: equivalent dosing volume measured into the beakers in mg/L; SD: standard deviation**

Table S4 aluminium sulphate dosages used during coagulation-flocculation experiments

| AS-HTW | | | AS-MTW | | | AS-LTW | | |
| --- | --- | --- | --- | --- | --- | --- | --- | --- |
| Dosage (mg/L) | Mean (n=3) | SD | Dosage (mg/L) | Mean (n=3) | SD | Dosage (mg/L) | Mean (n=3) | SD |
| 0.5 | 10 | 0.03 | 0.5 | 7.4 | 0.53 | 0.5 | 0.4 | 0.05 |
| 1.0 | 3.6 | 0.12 | 1.0 | 5.9 | 0.36 | 1.0 | 0.3 | 0.02 |
| 5.0 | 2.6 | 0.11 | 5.0 | 2.4 | 0.36 | 5.0 | 0.2 | 0.04 |
| 10 | 1.7 | 0.25 | 10 | 0.8 | 0.11 | 10 | 0.4 | 0.04 |
| 20 | 1.6 | 0.07 | 20 | 1.5 | 0.06 | 20 | 1.0 | 0.01 |
| 50 | 1.4 | 0.04 | 50 | 2.5 | 0.25 | 50 | 1.4 | 0.14 |

**SD: standard deviation**


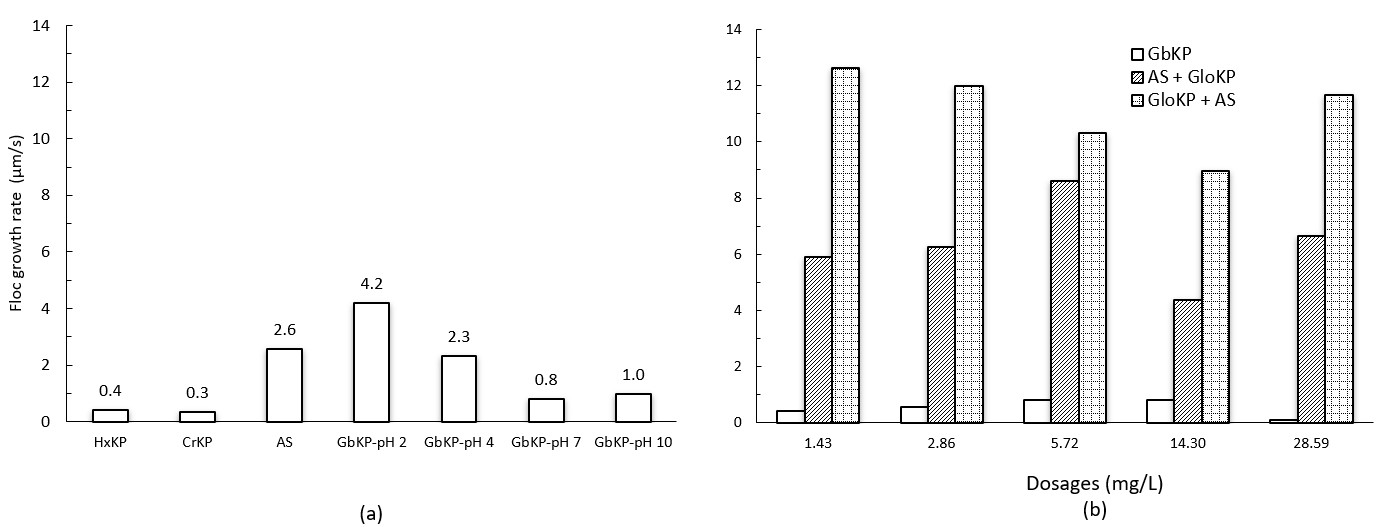


**Fig. S1 (a) floc growth rate of optimum dosages of HxKP, CrKP, AS and for different pH of GloKP. (b) the growth rate of AS, GloKP, GlobKP + AS and AS + GloKP after slow stir phase of 15mins. Operating conditions: turbidity (150 NTU), T=20^o^C.**
